# Distribution and current conservation status of the Mexican Goodeidae (Actinopterygii, Cyprinodontiformes)

**DOI:** 10.3897/zookeys.885.38152

**Published:** 2019-11-04

**Authors:** John Lyons, Kyle R. Piller, Juan Miguel Artigas-Azas, Omar Dominguez-Dominguez, Pablo Gesundheit, Michael Köck, Martina Medina-Nava, Norman Mercado-Silva, Arely Ramírez García, Kearstin M. Findley

**Affiliations:** 1 University of Wisconsin Zoological Museum, Madison, USA; 2 Southeastern Louisiana University, Hammond, USA; 3 San Luis Potosí, México; 4 Universidad Michoacana de San Nicolás de Hidalgo, Morelia, México; 5 Universidad Nacional Autónoma de México, Ciudad de México, México; 6 Haus des Meeres Aqua-Terra Zoo, Vienna, Austria; 7 Universidad Michoacana de San Nicolás de Hidalgo, Morelia, México; 8 Universidad Autónoma del Estado de Morelos, Cuernavaca, México; 9 Universidad Michoacana de San Nicolás de Hidalgo, Morelia, México; 10 Southeastern Louisiana University, Hammond, USA

**Keywords:** Captive maintenance, Evolutionarily Significant Unit, Goodeinae, Mexico

## Abstract

The current distribution and abundance of the 40 species of Goodeidae fishes known from Mexico are described, and a total of 84 Evolutionarily Significant Units (ESUs) is designated within these species. Two species and four ESUs are likely extinct with no captive populations, and three species and eight ESUs are probably extinct in the wild but have at least one captive population in Mexico, the United States, or Europe. Of the 35 extant species, the analyses indicate that nine should be considered as critically endangered, 14 as endangered, nine as vulnerable, and only three as least concern. Twenty-seven of these species have experienced substantial declines in distribution or abundance or both since 2000, and only eight appear to have remained relatively stable. Of the 72 extant ESUs, our analyses indicate that 29 should be considered as critically endangered, 21 as endangered, 18 as vulnerable, and only four as least concern. Brief summaries of the historic and current distributions and abundance of each species are provided, as well as ESU. Three strategies are recommended to conserve Mexican goodeids: protect the best-quality remaining habitats where goodeids still persist, restore degraded habitat and re-introduce species or ESUs where practical, and establish captive populations to ensure continued survival of the many species and ESUs that will almost inevitably go extinct in the coming years. Limited resources require cooperation and collaboration between scientists, conservationists, and aquarium hobbyists for successful captive maintenance.

## Introduction

**Figure 1.**
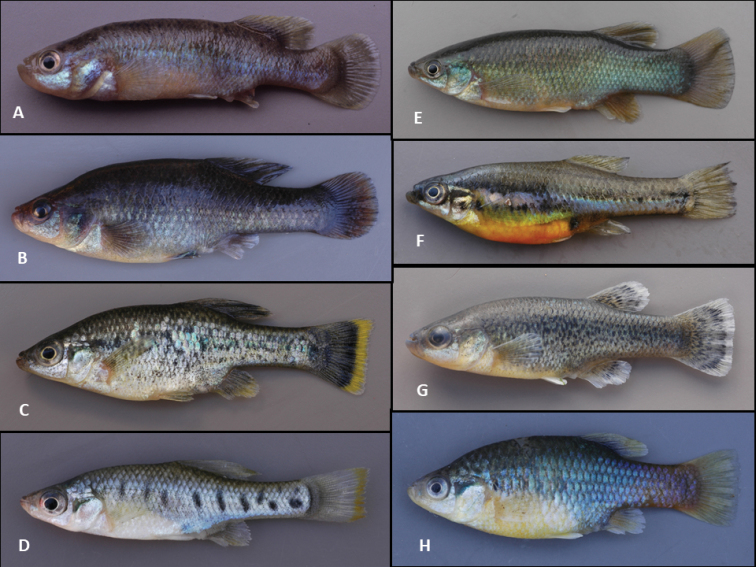
Photos of eight representative goodeid fishes. All photos taken by John Lyons of freshly preserved, wild-caught, adult, male specimens. **A***Allotoca goslinei*, Potrero Grande Stream, Jalisco, 8 January 2004 **B***Alloophorus robustus*, Opopeo Lake, Michoacán, 9 January 2011 **C***Ameca splendens*, Almoloya Springs, Jalisco, 17 January 2008 **D***Chapalichthys encaustus*, Lake Chapala near Ajijic, Jalisco, 6 January 2005 **E***Goodea atripinnis*, Tierra Quemada Stream, San Luis Potosí, 15 January 2011 **F***Skiffia lermae*, Zacapu Lake, Michoacán, 11 January 2005 **G***Xenotaenia resolanae*, Cuzalapa River, Jalisco, 10 January 2006 **H***Xenotoca doadrioi*, San Marcos Stream, Jalisco, 9 January 2005.

**Figure 2.**
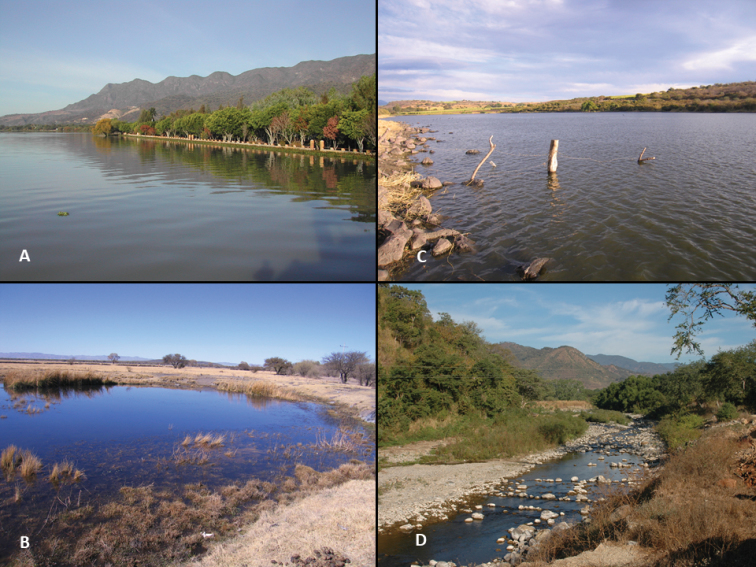
Photos of four representative types of goodeid habitats **A** Lake Chapala, near Ajijic, Jalisco, 6 January 2005. The largest natural lake in Mexico and historically the home of at least six species of goodeids. Currently, only *Chapalichthys encaustus* (see Figure 1), *Goodea atripinnis*, and *Zoogoneticus purhepechus* remain **B** 27 de Noviembre Springs, Durango, 11 January 2008, home of *Characodon* species **C** Molino Viejo Reservoir, Jalisco, 17 January 2008, home of *Xenotoca melanosoma***D** Cuzalapa River, Jalisco, 11 January 2006, home of *Ilyodon furcidens* and *Xenotaenia resolanae* (see Figure 1).

**Figure 3.**
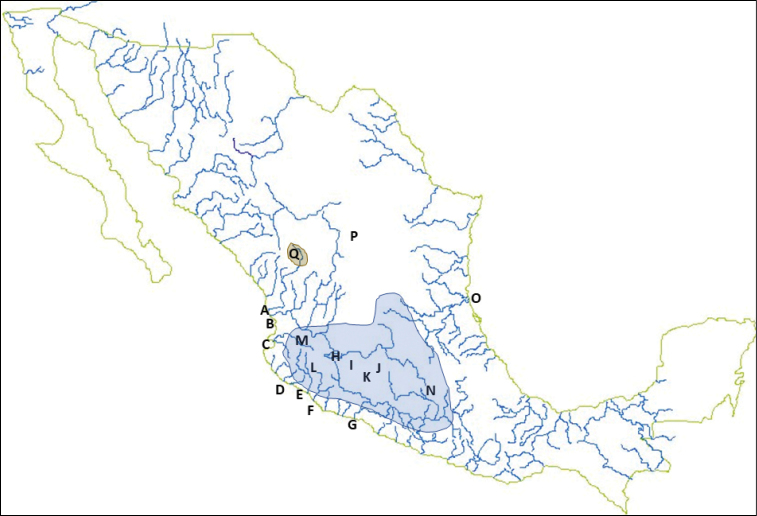
General distribution of goodeid fishes in Mexico, with the location of major river basins and lakes shown. Shaded areas indicate the range of most goodeid species (blue) and the disjunct range of *Characodon* (brown).**A** Mezquital River basin **B** Grande Santiago River Basin **C**Ameca River basin **D** Purificación and Marabasco River basins **E** Armería River basin **F** Coahuayana River basin **G** Balsas River Basin **H** Lake Chapala **I** Lerma River Basin **J** Lake Cuitzeo/Grande de Morelia River basin (endorheic) **K** Lake Pátzcuaro and Lake Zirahuén basins (endorheic) **L** Lake Atotonilco, Lake San Marcos, Lake Sayula, and Lake Zapotlán basins (endorheic) **M** Lake Magdalena basin (endorheic) **N** Valley of Mexico/ Mexico City (endorheic) **O** Pánuco River basin **P** Valley of Parras,(endorheic), *Characodon garmani* collection site **Q** Tunal River drainage (part of Mezquital River basin), home of *Characodon* species.

The Goodeidae (Pisces, Cyprinodontiformes) is a family of small-bodied freshwater fishes found in Mexico and the United States. There are two subfamilies, the Empetrichthyinae, with three oviparous species and multiple subspecies found in the Great Basin of the southwestern United States (Campbell and Piller 2017), and the Goodeinae, with ca. 40 viviparous species found in the highlands of central Mexico (Figures 1–3) (Parenti 1981). All Empetrichthyinae species and subspecies are either extinct, endangered, or threatened, and protection and recovery programs have been established for the few remaining populations (Jelks et al. 2008; Minckley and Marsh 2009). The state of the Goodeinae is less clear. A handful of species are clearly extinct or endangered and a few others are believed to be relatively stable and secure, but the current conservation status for many species is undefined and appears to be rapidly changing for the worse (Lyons et al. 1998; Contreras-Balderas et al. 2003; De la Vega-Salazar and Macías-Garcia 2005; Domínguez-Domínguez et al. 2005a, b; Jelks et al. 2008; Ramírez-Carrillo and Macías-García 2015; Gesundheit and Macías-García 2018).

The distribution and abundance of most species within the Goodeidae has declined precipitously during the last 20 years, and the continued survival of many species has become precarious (Lyons et al. 1998; Soto-Galera et al. 1998, 1999; De la Vega-Salazar et al. 2003a; Domínguez-Domínguez et al. 2008a; Helmus et al. 2009; Ramírez-Carrillo and Macías-García 2015). Within some species, unique evolutionary lineages, which are just now being identified and delineated, are on the brink of elimination. There is an urgent need to better document these lineages and their native ranges (De la Vega-Salazar et al. 2003b; Medina-Nava et al. 2005, Domínguez-Domínguez et al. 2006; Helmus et al. 2009; Lyons 2013). For some species and lineages, the situation in the wild is so dire that captive maintenance and breeding programs must be implemented as soon as possible to avoid their imminent extinction (Bailey et al. 2007; Domínguez-Domínguez et al. 2008a; Lyons 2013; Maceda-Veiga et al. 2014).

In this paper, we have assessed the current (2019) conservation status of goodeid fishes in the wild in Mexico (Goodeinae), updating and expanding upon the status and trends surveys from Domínguez-Domínguez et al. (2005a, b), which were published in a book that is not readily available, and the non-peer-reviewed popular summary in Lyons (2013), which was published in an aquarium hobbyist book in German. We have focused on documenting unique evolutionary lineages within populations that are worthy of conservation, which we have termed Evolutionarily Significant Units or ESUs. We have followed the definition of ESUs given by Crandall et al. (2000), which incorporates both genetic and ecological distinctiveness and adaptive significance. We propose that this ESU concept will provide a helpful framework for developing protection and restoration plans for wild populations and for compiling priority lists and husbandry guidelines for captive populations. Designation of ESUs may also focus attention on unique populations that may eventually be described as new species. The ESU concept was first applied for fish to Pacific salmon (*Oncorhynchus* species), in which discrete spawning runs were designated as separate ESUs if they met the criteria of being largely reproductively isolated from other potential ESUs and also constituting important components of the evolutionary legacy of the species (Waples 1991), criteria that also apply to the goodeid ESUs we have designated. The ESU concept has since become an important component of the conservation framework for many different fish species (e.g., Hedrick et al. 2001; Stockwell et al. 2013).

## Materials and methods

We have determined the conservation status of the Mexican goodeids based on a combination of the recent scientific literature, museum specimens, communication with other aquatic scientists, and especially our own personal field collections, with emphasis on surveys conducted within the last 20 years. We have generally followed the taxonomy proposed by Miller et al. (2005) but have made some modifications (noted in the text) based on recent genetic analyses by Doadrio and Domínguez-Domínguez (2004) and Webb et al. (2004). We have also included new species described by Radda and Meyer (2003) and Domínguez-Domínguez et al. (2008b, 2016). Collectively, since 2000, we have sampled all known and likely locations for every distinctive population of all of the nominal Mexican goodeid species.

We have defined ESUs based on genetic, morphological, and zoogeographic evidence for distinctive population structure within each goodeid species. All of our proposed ESUs are or were allopatric from each other, occurring within discrete river basins or sub-basins. Genetic evidence for ESUs was based primarily on sequence divergence within the mitochondrial cytochrome b gene, the genetic marker most commonly used thus far in Mexican goodeid studies. We considered sequence divergence of 1% (uncorrected p-distance) or more between populations as sufficient to designate ESUs because many of the species described prior to the inclusion of genetic data are at least 1% divergent from their putative sister species. Where no genetic divergence was evident or where genetic data were lacking, we also relied on morphological analyses to identify ESUs. We required statistically significant multivariate morphological or meristic differences with limited overlap among populations to designate ESUs. Finally, where both genetic and morphological data were lacking, we employed zoogeographic information to designate ESUs, relying heavily on Domínguez-Domínguez et al. (2006a). In this case, we identified ESUs if geological evidence indicated that populations in different basins had likely been isolated for at least 50,000 years and other fish taxa showed clear genetic or morphological differences between the basins. We developed an alphanumeric code to identify ESUs, consisting of three letters from the genus name followed by two letters of the species name followed by a unique number for the ESU. For example, the four ESUs we identified for *Alloophorus robustus* were assigned the codes Alpro1, Alpro2, Alpro3, and Alpro4.

We determined the conservation status of goodeid species and ESUs based on the terminology and criteria of the International Union for the Conservation of Nature (IUCN 2012). Two of the authors of this paper, Omar Domínguez-Domínguez and Michael Köck, participated in an IUCN-sponsored workshop in December 2018 to assess the conservation status of Mexican freshwater fishes. This resulted in most of our status designations for goodeid species being adopted by the IUCN, and these will be published by IUCN in 2019. However, in five cases there were inconsistencies between our and the IUCN’s designations, most prominently in that IUCN did not complete assessments for *Girardinichthys ireneae*, *G. turneri*, *Xenotoca doadrioi*, and *X. lyonsi*. The IUCN also considers the re-introduced population of *Zoogoneticus tequila* as established and gave it a status of “Endangered” while we still considered it “Extinct in the Wild” because we felt it was too early to declare the population fully re-established. The IUCN workshop also did not assess potential ESUs.

The IUCN criteria we used are as follows. “Extinct” indicated species or ESUs for which no specimens have been collected despite repeated targeted surveys in appropriate habitats. We distinguished between species or ESUs for which no living specimens existed anywhere on earth (“extinct”) and those for which no specimens occurred in nature but for which viable captive populations were still present (“extinct in the wild”). “Critically endangered” was applied to species or ESUs that either persisted in the wild at only 1–3 distinct locations with an estimated combined minimum annual population of fewer than 250 breeding adults or that had experienced overall decreases in distribution and abundance of more than 80% within the last ten years. “Endangered” species or ESUs either occurred at 4–8 distinct locations with a combined minimum annual population of no more than 2,500 breeding adults or had decreased in distribution and abundance by 50–70% within the last ten years. “Vulnerable” species or ESUs either occurred at 9–35 distinct locations with a combined minimum annual population of no more than 10,000 breeding adults or had declined by 30–50% in distribution and abundance within the last ten years. “Nearly threatened” species or ESUs were uncommon and in decline, but they did not quite meet the criteria for designation as vulnerable or endangered. “Least concern” species or ESUs had a broader and more stable distribution and abundance and were not in immediate danger of being designated as vulnerable or endangered, although they may have been declining in distribution or abundance in some areas.

We have also reported the official Mexican government status designation from the federal regulations established to protect rare species (“Norma Oficial Mexicana”; NOM-059-SEMARNAT-2010; NOM 2010 hereafter). Four categories have been applied to the goodeids: “Extinct” (Extinto) – no specimens encountered despite repeated targeted sampling of appropriate habitats; “Endangered” (En Peligro) – species rare and in decline and likely to become extinct within the near future without protection and management; “Threatened” (Amenazado) – species uncommon and in decline and likely to become endangered within the near future without protection and management, and “Under Special Protection” (Sujeta Protección Especial) – species in decline and needing regulation although not qualifying as threatened or endangered.

We have provided population trends for each species based upon our and colleagues’ observations (mostly unpublished) since ca. 2000. If we noted the disappearance of a species from one or more locations or the substantial decline in abundance of one or more populations, we classified the species as declining. If no populations had been eliminated and abundances showed no clear trend, we classified the species as stable. If the species was expanding its range through movement and colonization of new habitats or if one or more populations had grown noticeably, we classified the species as increasing. Note that an increase in the range of a species based on the discovery of a new population that was believed to always have been present did not qualify a species to be classified as increasing.

## Results

### Summary of goodeid conservation status and population trends

**Figure 4.**
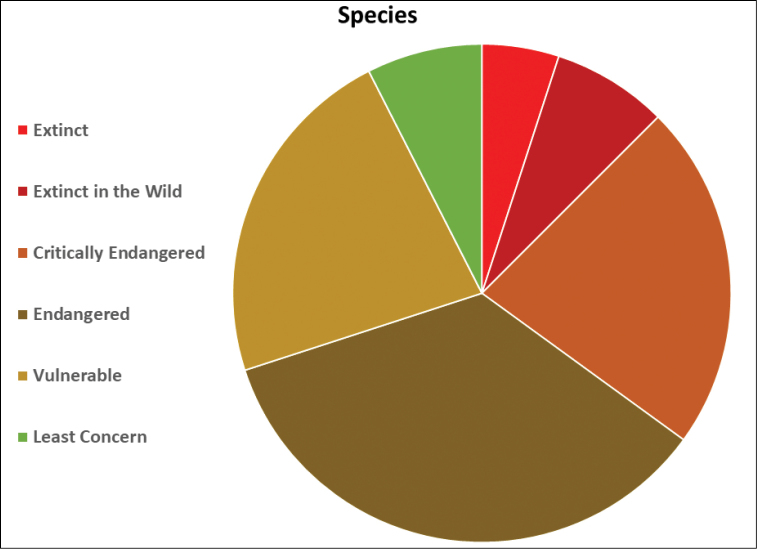
Pie chart of the conservation status of Mexican goodeid species.

Nearly all Mexican goodeids qualified for a protected conservation status designation under the IUCN criteria (Table 1). Of the 40 species we recognized, two were extinct, three were extinct in the wild, nine were critically endangered, 14 were endangered, nine were vulnerable, none were nearly threatened, and only three were least concern (Figure 4). The Mexican government (NOM 2010), which used a somewhat different taxonomic classification from ours and did not recognize several recently described species, officially listed one species as extinct, 18 as endangered, four as threatened, and one under special protection. Most of the government designations generally agreed with ours with two major exceptions: classifying *Ilyodon furcidens* as threatened whereas we classified it as least concern, and giving no formal protected status to *Allotoca maculata*, *A. meeki*, and *Chapalichthys pardalis*, which we classified as critically endangered, and *Girardinichthys multiradiatus*, which we classified as endangered. Of the 35 species we recognized that were still found in nature, we determined that 27 were declining and only eight were stable. Three species have recently been introduced by humans into new drainage basins, *Chapalichthys encaustus* into the Ameca River basin in the state of Jalisco, *Goodea atripinnis* into the Mezquital River basin in the state of Durango, and *Ilyodon furcidens* into the Citala Reservoir in the Lake Sayula basin in Jalisco, but these expansions have been offset by declines and losses of populations within their native ranges. Therefore, no species qualified as increasing.

**Table 1.** Status and trends of Mexican Goodeidae in the wild as of 2019. “Mexico” refers to the legal classification by the Mexican Federal government (NOM 2010; NC Not Classified; Sp. Protec. Under Special Protection), and “This study” refers to the IUCN designation based on our analyses.

**Species**	**Conservation Status**		**Trend Since 2000**
	**Mexico**	**This Study**	
*Allodontichthys hubbsi*	Endangered	Endangered	Stable
*Allodontichthys polylepis*	Endangered	Critically Endangered	Declining
*Allodontichthys tamazulae*	Endangered	Vulnerable	Stable
*Allodontichthys zonistius*	None	Vulnerable	Stable
*Alloophorus robustus*	None	Vulnerable	Declining
*Allotoca catarinae*	Endangered	Vulnerable	Stable
*Allotoca diazi*	Endangered	Critically Endangered	Declining
*Allotoca dugesii*	Endangered	Endangered	Declining
*Allotoca goslinei*	Endangered	Extinct in the wild?	No records since 2004
*Allotoca maculata*	None	Critically Endangered	Declining
*Allotoca meeki*	None	Critically Endangered	Declining
*Allotoca zacapuensis^1^*	NC	Critically Endangered	Stable
*Ameca splendens*	Endangered	Endangered	Declining
*Ataeniobius toweri*	Endangered	Endangered	Declining
*Chapalichthys encaustus*	None	Vulnerable	Declining
*Chapalichthys pardalis*	None	Critically Endangered	Declining
*Characodon* species	Endangered	Critically Endangered	Declining
*Characodon garmani^1^*	NC	Extinct	No records since 1890’s
*Girardinichthys ireneae^1^*	NC	Critically Endangered	Declining
*Girardinichthys multiradiatus*	None	Endangered	Declining
*Girardinichthys turneri*	Endangered	Extinct?	No records since 1980’s
*Girardinichthys viviparus*	Endangered	Endangered	Stable
*Goodea atripinnis*	None	Least Concern	Declining
*Ilyodon furcidens*	Threatened	Least concern	Declining
*Ilyodon whitei*	None	Vulnerable	Declining
*Neoophorus regalis*	Endangered	Critically Endangered	Declining
*Neotoca bilineata*	Endangered	Endangered	Declining
*Skiffia francesae*	Extinct	Extinct in the wild	No records since 2008
*Skiffia lermae*	Threatened	Endangered	Declining
*Skiffia multipunctata*	Threatened	Endangered	Declining
*Xenoophorus captivus*	Endangered	Endangered	Declining
*Xenotaenia resolanae*	None	Vulnerable	Stable
*Xenotoca doadrioi^1^*	NC	Endangered	Declining
*Xenotoca eiseni*	Sp. Protec.	Endangered	Declining
*Xenotoca lyonsi^1^*	NC	Endangered	Declining
*Xenotoca melanosoma*	Endangered	Vulnerable	Declining
*Xenotoca variata*	None	Least Concern	Declining
*Zoogoneticus purhepechus^1^*	NC	Vulnerable	Declining
*Zoogoneticus quitzeoensis*	Threatened	Endangered	Declining
*Zoogoneticus tequila*	Endangered	Extinct in the wild^2^	No records since 2008^2^

^1^ These six species were not recognized and hence not classified by the Mexican government.

^2^*Zoogoneticus tequila* was considered extinct in the wild as of 2008, but in 2016 it was re-introduced and has successfully reproduced. It is too early to determine whether it will become permanently re-established.

**Figure 5.**
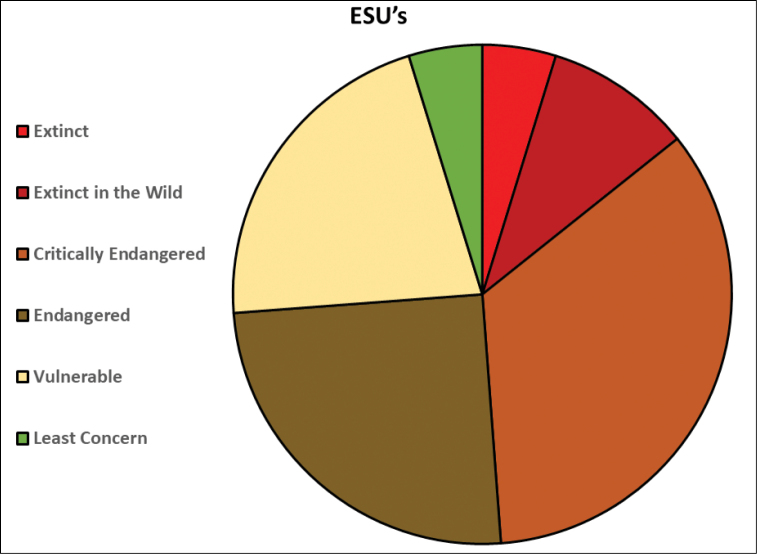
Pie chart of the conservation status of Mexican goodeid evolutionarily significant units (ESUs).

For the 40 goodeid species, we have identified 84 ESUs (Table 2). The number of ESUs per species varied substantially, with 20 species with only one ESU, 10 species with two, three species with three, four species with four, two species with five, and one species with nine. Most ESUs qualified for a protected conservation designation. Of the 84 ESUs, we determined that four were likely extinct, eight extinct in the wild, 29 critically endangered, 21 endangered, 18 vulnerable, none nearly threatened, and four least concern (Figure 5).

**Table 2.** Number of proposed Evolutionarily Significant Units (ESUs) per goodeid species and their conservation status (IUCN 2012). Key: EX Extinct, EW extinct in the wild, CE critically endangered, EN endangered, VU vulnerable, NT nearly threatened, and LC least concern.

Species	Number of ESUs	Conservation status						
		EX	EW	CE	EN	VU	NT	LC
*Allodontichthys hubbsi*	2	0	0	1	1	0	0	0
*Allodontichthys polylepis*	1	0	0	1	0	0	0	0
*Allodontichthys tamazulae*	1	0	0	0	0	1	0	0
*Allodontichthys zonistius*	1	0	0	0	0	1	0	0
*Alloophorus robustus*	4	0	0	2	0	2	0	0
*Allotoca catarinae*	1	0	0	0	0	1	0	0
*Allotoca diazi*	1	0	0	1	0	0	0	0
*Allotoca dugesii*	4	0	0	3	1	0	0	0
*Allotoca goslinei*	1	0	1	0	0	0	0	0
*Allotoca maculata*	2	0	0	2	0	0	0	0
*Allotoca meeki*	1	0	0	1	0	0	0	0
*Allotoca zacapuensis*	1	0	0	1	0	0	0	0
*Ameca splendens*	3	0	1	2	0	0	0	0
*Ataeniobius toweri*	1	0	0	0	1	0	0	0
*Chapalichthys encaustus*	1	0	0	0	0	1	0	0
*Chapalichthys pardalis*	2	0	0	2	0	0	0	0
*Characodon* species	9	1	2	6	0	0	0	0
*Characodon garmani*	1	1	0	0	0	0	0	0
*Girardinichthys ireneae*	1	0	0	1	0	0	0	0
*Girardinichthys multiradiatus*	2	0	0	0	2	0	0	0
*Girardinichthys turneri*	1	1	0	0	0	0	0	0
*Girardinichthys viviparus*	1	0	0	0	1	0	0	0
*Goodea atripinnis*	2	0	0	0	1	0	0	1
*Ilyodon furcidens*	1	0	0	0	0	0	0	1
*Ilyodon whitei*	5	0	0	0	0	5	0	0
*Neoophorus regalis*	1	0	0	1	0	0	0	0
*Neotoca bilineata*	2	1	0	0	1	0	0	0
*Skiffia francesae*	2	0	2	0	0	0	0	0
*Skiffia lermae*	4	0	0	1	3	0	0	0
*Skiffia multipunctata*	1	0	0	0	1	0	0	0
*Xenoophorus captivus*	3	0	1	1	1	0	0	0
*Xenotaenia resolanae*	2	0	0	0	0	2	0	0
*Xenotoca doadrioi*	1	0	0	0	1	0	0	0
*Xenotoca eiseni*	2	0	0	0	2	0	0	0
*Xenotoca lyonsi*	1	0	0	0	1	0	0	0
*Xenotoca melanosoma*	4	0	0	1	2	1	0	0
*Xenotoca variata*	5	0	0	0	0	3	0	2
*Zoogoneticus purhepechus*	3	0	0	1	1	1	0	0
*Zoogoneticus quitzeoensis*	2	0	0	1	1	0	0	0
*Zoogoneticus tequila*	1	0	1	0	0	0	0	0
Totals	84	4	8	29	21	18	0	4

### Conservation status and populations trends of species

***Allodontichthys***: This genus consists of four bottom-dwelling species found in fast-flowing areas of streams and rivers on the Pacific slope of west-central Mexico in the Ameca, Armería, and Coahuayana river basins (Lyons and Mercado-Silva 2000; Webb 2002). It is most closely related to *Ilyodon* and *Xenotaenia* (Webb 2002; Doadrio and Domínguez-Domínguez 2004; Webb et al. 2004).

**Figure 6.**
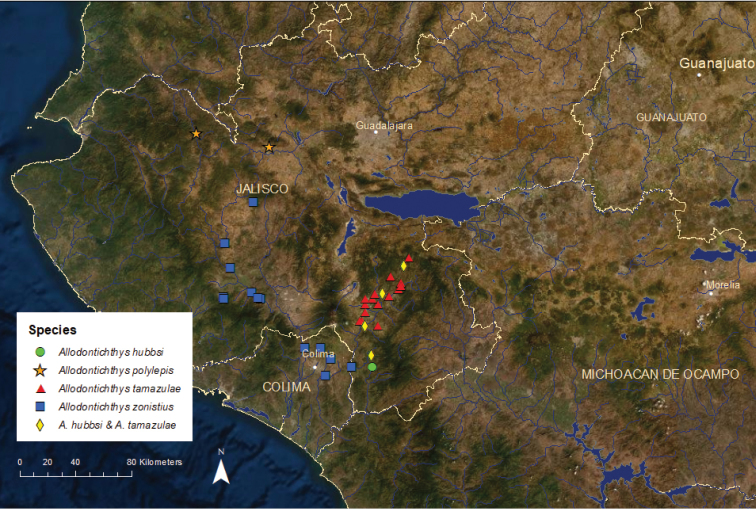
Distribution of the four species of *Allodontichthys*.

*Allodontichthys hubbsi*: Endangered/Stable/2 ESUs (Figure 6) – This species is known from only six areas in the upper Coahuayana River basin (Lyons and Mercado-Silva 2000). We recognized two ESUs based on genetic analyses (Domínguez unpublished data). Aldhu1 is endangered and occupies four areas of the Tamazula River drainage, short segments of the Tamazula River, the San Jerónimo River, a small unnamed tributary of the Tamazula, and the Contla Stream. The Contla Stream had the best population with several hundred adults. Aldhu2 is critically endangered and has small populations in three tributaries of the Coahuayana River that are isolated from the Tamazula River by waterfalls, San Jose del Tule Stream, El Terrero Stream, and Pihuamo River. A 2019 survey found dozens of individuals in the San Jose del Tule, a single individual in the El Terrero, and none at the one site sampled on the Pihuamo.

*Allodontichthys polylepis*: Critically Endangered/Declining/1 ESU (Aldpo1) (Figure 6) – As of its initial description in 1988 (Rauchenberger 1988), this species was known from three locations in the upper Ameca River basin. By 2000, the Potrero Grande Stream population had disappeared for unknown reasons, but the populations in the De la Pola River (reported as Bola by some collectors) and its tributary the Dávalos Stream (reported as Diábalos by some collectors) had persisted (Domínguez-Domínguez et al. 2005b). These two populations have declined since then, and an intensive 2016 survey yielded only a single individual from each water body.

*Allodontichthys tamazulae*: Vulnerable/Stable/1 ESU (Aldta1) (Figure 6) – Historically known from throughout the Upper Coahuayana River basin where it coexisted with *A. hubbsi* (Miller et al. 2005). Pollution from a sugar cane mill near the town of Tamazula has made a portion of the former range of the species in the lower Tamazula River uninhabitable since the 1970s (Lyons and Mercado-Silva 2000). Our recent surveys have encountered *A. tamazulae* at ten locations, several of which had moderately large numbers of fish, and populations appear to be stable.

*Allodontichthys zonistius*: Vulnerable/Stable/1 ESU (Aldzo1) (Figure 6) – Known from 12 locations in the Armería River basin and two nearby areas in the lower Coahuayana River basin, which originated from a relatively recent stream capture (Lyons and Mercado-Silva 2000; Webb 2002). Improved water quality from a sugar mill discharge has led to increases in abundance in the Ayuquila River in the Armería basin near the city of Autlán, but these gains have been offset by population declines further downstream in the Armería River and its tributaries near the city of Colima.

***Alloophorus***: This genus contains a single species, *A. robustus*, which historically was widespread in the Lerma, upper Santiago (including Lake Chapala), and upper Balsas river basins on the Pacific slope and the endorheic (no outlet) Lakes Pátzcuaro and Zirahuén, and Lake Cuitzeo/Grande de Morelia River basins in central Mexico (Miller et al. 2005). The species has broad habitat tolerances, occurring in lakes, springs, and rivers. As a result of its relatively large body size, in the past, *A. robustus* was harvested for human food in many places, although currently most populations are too small to support a significant fishery. The species is still harvested in Lake Pátzcuaro and Lake Zacapu, Michoacán.

**Figure 7.**
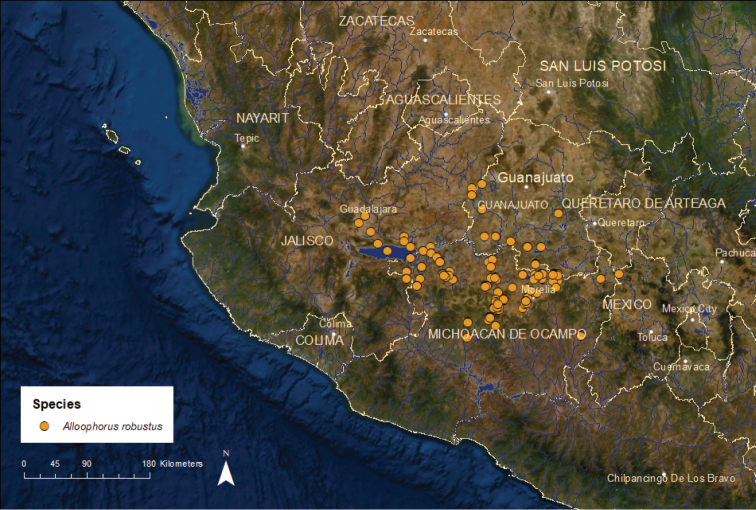
Distribution of *Alloophorus robustus*.

*Alloophorus robustus*: Vulnerable/Declining/4 ESUs (Figure 7) – Once known from more than 50 different localities, this species now persists at approximately 25 localities. Since 2000, it has disappeared from Lake Chapala and the adjacent Santiago and Lerma rivers, from the De la Laja River, a major Lerma tributary, and from Lake Yuriria. The species has become rare in the Lake Cuitzeo/Grande de Morelia River, Lake Pátzcuaro and Lake Zirahuén basins, persisting mainly in heavily vegetated lake shorelines, spring areas, and small tributaries (Lyons et al. 1998; Soto-Galera et al. 1998, 1999; Mercado-Silva et al. 2006; Domínguez-Domínguez et al. 2008b). Losses have been from a combination of declines in water quality and quantity (e.g., Chapala, Cuitzeo) and predation and competition from introduced non-native species (e.g., *Xiphophorus variatus* (Poeciliidae) in the De la Laja River; *Micropterus salmoides* (Centrarchidae) in Lake Zirahuén). Remaining strongholds include the La Mintzita Springs in the Lake Cuitzeo basin near the city of Morelia, Lake Zacapu in the headwaters of the Angulo River drainage, a Lerma River tributary, and the Duero River drainage, also a Lerma River tributary, including the La Luz and Orandino spring-fed lakes. We recognize four ESUs based on genetic analyses and biogeography. Alpro1 is vulnerable and occupies much of the Lerma River basin (excluding the Turbio River drainage) and the upper Balsas River basin, Alpro2 is vulnerable and occurs in the Lake Cuitzeo/Grande de Morelia River basin, Alpro3 is critically endangered and is found at only one or two sites in the Turbio River drainage in the Lerma River basin, and Alpro4 is critically endangered and known from the Lake Pátzcuaro and Lake Zirahuén basins. It persists at just one or two locations in small numbers in the Pátzcuaro basin.

***Allotoca***: This is the most diverse genus of goodeids, with seven currently recognized species and probably one or more additional undescribed species (Doadrio and Domínguez-Domínguez 2004; Webb et al. 2004; Miller et al. 2005). Overall, the genus had a historically wide range in the Ameca, Armería, Magdalena, Lerma, Cuitzeo/Grande de Morelia, Pátzcuaro, Zirahuén, and upper Balsas river basins on the Pacific slope of central Mexico. However, the individual range of most species is (and might have always been) quite small. Some populations of *Allotoca* that have disappeared may have represented additional undescribed species. For example, a single distinctive *Allotoca* specimen was collected from the upper Armería River basin in the 1930s and a different but also distinctive *Allotoca* specimen was found in Lake Chapala in the 1960s, but no *Allotoca* have been found in either location since. Thus, whatever species these specimens represented, new or otherwise, they have been extirpated (Lyons et al. 1998), and they are not counted in any of our totals or summaries.

**Figure 8.**
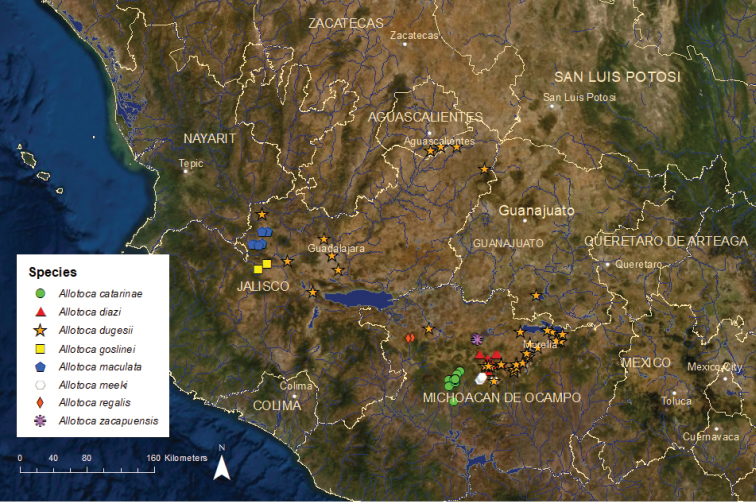
Distribution of the eight species of *Allotoca*.

*Allotoca catarinae*: Vulnerable/Stable/1 ESU (Altca1) (Figure 8) – This species is known from approximately ten locations in the upper Cupatitzio River drainage in the upper Balsas River basin near the city of Uruapan and possibly also in the Lake Cuitzeo/Grande de Morelia and Lake Pátzcuaro basins, although the taxonomic status of specimens from outside of the Cupatitzio River drainage is uncertain (Doadrio and Domínguez-Domínguez 2004). None of these populations are particularly large, but all have persisted since 2000. This species is genetically very similar to *A. diazi* and may have reached the upper Balsas basin by a human transfer within the last 1,000 years (Corona-Santiago et al. 2015).

*Allotoca diazi*: Critically Endangered/Declining/1 ESU (Altdi1) (Figure 8) – Older literature (e.g., Meek 1904; Mendoza 1962) placed this species in the genus *Neoophorus*, but recent morphological and genetic analyses indicate that *Allotoca* is more appropriate (Meyer et al. 2001; Doadrio and Domínguez-Domínguez 2004; Webb et al. 2004). Currently, *A. diazi* is known from just three small areas in the Lake Pátzcuaro basin, the only basin where it occurs. It has declined dramatically in Lake Pátzcuaro proper and persists only as a remnant population there. The largest remaining population is in the Molino de Chapultepec Springs near the town of Pátzcuaro.

*Allotoca dugesii* (spelled *dugesi* in older literature; e.g., Smith and Miller 1980): Endangered/Declining/4 ESUs (Figure 8) – The widest ranging of the *Allotoca* species, historically known from much of the middle and lower Lerma and upper Santiago river basins on the Pacific slope and the endorheic Lake Pátzcuaro, Lake Zirahuén, and Lake Cuitzeo/Grande de Morelia River basins (Miller et al. 2005). Currently, the species is known from only six or seven locations. A new population was recently discovered in a spring along the Duero River in the Lerma River basin near the town of Etúcuaro, Michoacán, but it is very small. Recent surveys have documented the species’ dramatic reduction in the upper Santiago basin and elimination from the Zirahuén basin where it was once widespread and common. Remaining strongholds are the Molino de Chapultepec Springs in the Lake Pátzcuaro basin and La Maiza Springs in the Cuitzeo/Grande de Morelia basin near the city of Morelia. Domínguez-Domínguez et al. (2002) published observations on larval feeding of this species that will be useful in the maintenance of captive populations. We recognized four ESUs based on zoogeography (Domínguez-Domínguez et al. 2006a, b). Altdu1 is critically endangered and is known from the upper Santiago River basin, Lake Chapala, and the lower Lerma River basin. However, perhaps only one viable population remains, in a spring near Lake Chapala. Altdu2 is critically endangered and found at a single site in the Turbio River drainage of the middle Lerma River basin. Atldu3 is endangered and occurs at 3–4 sites in the Lake Cuitzeo/Grande de Morelia River basin. Altdu4 is critically endangered and known historically from the Lake Pátzcuaro and Lake Zirahuén basins. Presently, it persists only along at a heavily vegetated shoreline area of southwestern Lake Pátzcuaro and in the Molino de Chapultepec Springs, a tributary of Lake Pátzcuaro.

*Allotoca goslinei*: Extinct in the Wild/No records since 2004/1 ESU (Altgo1) (Figure 8) – This species was known from only a small tributary of the Ameca River, the Potrero Grande Stream, and the Ameca River itself near the mouth of the stream in the upper Ameca River basin near the city of Ameca (Smith and Miller 1987). The species had been eliminated from the Ameca River by the 1990’s by water pollution but was still moderately common in the headwaters of the Potrero Grande Stream. In the early 2000’s, the non-native *Xiphophorus helleri* (Poeciliidae) became established in the Potrero Grande Stream. As *X. helleri* numbers increased, the abundance of *A. goslinei* dropped rapidly, presumably from competition or predation on larvae. The last specimen of *A. goslinei* was collected in 2004, and none could be found in targeted surveys in 2005, 2006, 2007, 2016, and 2018 (Helmus et al. 2009; Köck unpublished data). Based on this, *A. goslinei* is feared to be extinct in the wild, although it is possible that a small population persists in an isolated area of the stream not yet invaded by *X. helleri*. Only a few captive populations exist in Mexico, the United States, and Europe, and all are small.

*Allotoca maculata*: Critically Endangered/Declining/2 ESUs (Figure 8) – This species was described from the endorheic Lake Magdalena basin in west-central Mexico, where it was thought to be endemic (Smith and Miller 1980). Believed extinct by the late 1980s (Miller et al. 1989), this species was rediscovered in the 1990s at two locations, Lake Magdalena and the nearby but hydrologically isolated headwaters of the San Marcos River in the Ameca River basin near the town of Etzatlán. Genetic analyses indicated that the San Marcos populations represented at least a distinct ESU and possibly an undescribed species (Doadrio and Domínguez-Domínguez 2004). We recognize two ESUs. Altma1, the Lake Magdalena ESU, is critically endangered and in decline from water pollution, habitat destruction, and non-native species. It has not been collected in more than four years despite several targeted surveys; and Altma2, the San Marcos ESU, is also critically endangered and in decline from water scarcity, habitat destruction, and non-native species, persisting in small numbers at one or two locations.

*Allotoca meeki*: Critically Endangered/Declining/1 ESU (Altme1) (Figure 8) – This species is known only from the endorheic Lake Zirahuén basin, where it was once common. The introduction of non-native *Micropterus salmoides* (Centrarchidae), a fish predator, eliminated the species from Lake Zirahuén by the late 1990’s (Domínguez-Domínguez et al. 2005b). A population of *A. meeki* persisted in Lake Opopeo in the headwaters of a tributary system, but by the 2000’s *M. salmoides* had invaded this lake, and *A. meeki* had become scarce. A small population has persisted in a short segment of the outlet of the lake, which appears to be too narrow and shallow for *M. salmoides*.

*Allotoca zacapuensis*: Critically Endangered/Stable/1 ESU (Altza1) (Figure 8) – This species was described in 2001 (Meyer et al. 2001) and is known only from Lake Zacapu and a tributary spring, Jesus Maria, which are in the headwaters of the Angulo River drainage in the Lerma River basin. Within the lake it is known from only two spring-fed areas where it is uncommon but apparently relatively stable in numbers (Domínguez-Domínguez et al. 2005b).

***Ameca***: This genus consists of a single species, *A. splendens*, which, until recently was thought to be restricted to the Teuchitlán Springs and their outlet in the upper Ameca River basin on the Pacific slope of west-central Mexico (Miller et al. 2005). However, within the last fifteen years, two new populations have been discovered in nearby basins.

**Figure 9.**
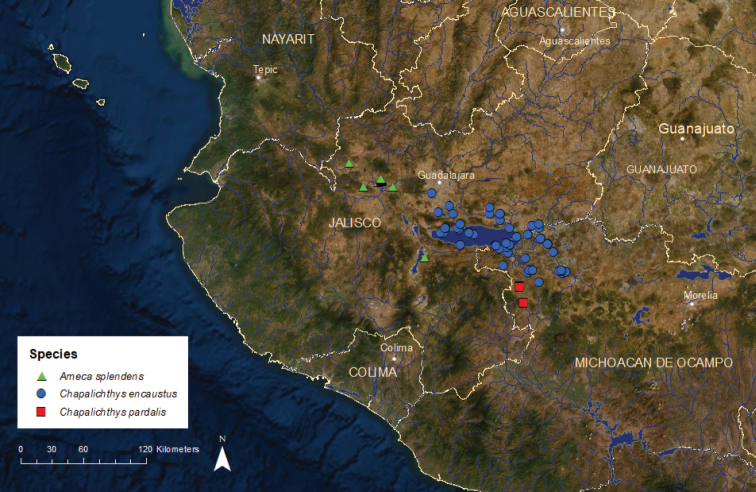
Distribution of *Ameca splendens*, *Chapalichthys encaustus*, and *C. pardalis*.

*Ameca splendens*: Endangered/Declining/3 ESUs (Figure 9) – This species is known from the Teuchitlán Springs and was recently discovered in the Almoloya Springs in the endorheic Lake Magdalena basin and the El Molino Springs in the endorheic Lake Sayula basin, both close to but separate from the Ameca basin. No genetic or morphological analyses of the three populations are available, but we consider each to be a separate ESU based on zoogeography. Amesp1 is endangered and occupies the Teuchitlán Springs and its outlet the Teuchitlán River. Historically, the population extended well down the river to its junction with the Salado River to form the Ameca River. However, it is now limited to the upper part of the Teuchitlán Springs and two small tributary springs that enter the river 2–3 km downstream. The Teuchitlán Springs population probably numbers in the high hundreds or low thousands and appears to be stable (López-López et al. 2004), but the two smaller springs have not been thoroughly assessed. Bailey et al. (2007) provided a detailed analysis of genetic diversity within the Teuchitlán Springs population. Amesp2 is critically endangered and known only from the Almoloya Springs in the Magdalena basin ca. 50 km northwest of Teuchitlán. The population has declined steadily since its discovery in the early 2000’s. The population decline was associated with the appearance and rapid population growth of non-native *Pseudoxiphophorus bimaculatus* (Poeciliidae), which is known to outcompete and threaten the survival of other goodeid species (Ramírez-Carrillo and Macías-García 2015). No Amesp2 specimens are held in captivity. Amesp3 is possibly extinct and known only from the El Molino Springs near Cuyucapán in the Sayula basin, ca. 80 km southeast of Teuchitlán. The population disappeared when the springs completely dried in 2010 during a drought. No Amspe3 specimens are in captivity. There are unconfirmed reports of populations of *Ameca splendens* from three other springs in the Sayula basin during the 1990’s (Rosales-Figueroa 1995), but these springs are now completely dry.

***Ataeniobius***: This genus is represented by a single species, *A. toweri*, and is limited to the thermal-spring lakes, springs, and streams in the headwaters of the Verde River drainage in the Pánuco River basin on the Atlantic slope of central Mexico (Miller et al. 2005).

**Figure 10.**
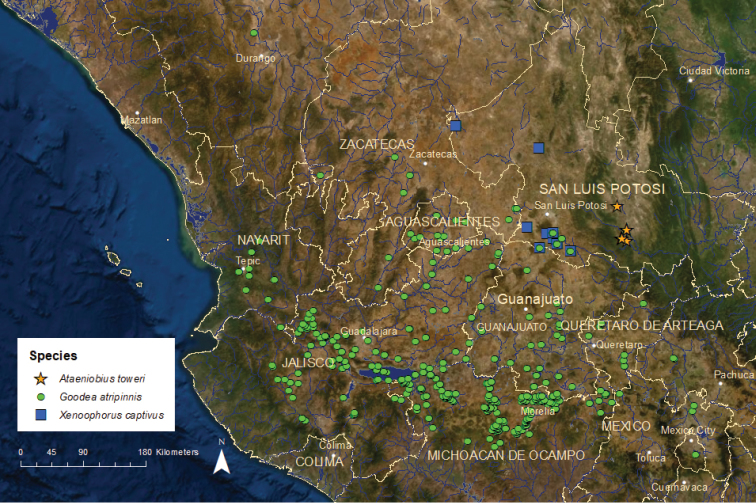
Distribution of *Ataeniobius toweri*, *Goodea atripinnis*, and *Xenoophorus captivus*.

*Ataeniobius toweri*: Endangered/Declining/1 ESU (Atato1) (Figure 10) – This species is known from the Media Luna and Los Anteojitos lakes, adjacent springs, their outlets near the city of Rioverde, and the Villa Juarez stream near the town of the same name (Domínguez-Domínguez et al. 2005b). *Ataeniobius toweri* associates closely with dense aquatic vegetation, and the recent loss of major macrophyte beds in Media Luna and Los Anteojitos have resulted in a substantial decline in the species abundance. At least two small nearby springs, Charco Azul and Los Peroles, maintain good populations. The Villa Juarez population persists but appears to be small.

***Chapalichthys***: This genus has two currently recognized species, one in the Lake Chapala basin and the other in a nearby portion of the upper Balsas River basin, both on the Pacific slope of central Mexico (Miller et al. 2005).

*Chapalichthys encaustus*: Vulnerable/Declining/1 ESU (Chaen1) (Figure 9) – This species was formerly abundant throughout nearshore areas of Lake Chapala and was also encountered in adjacent areas of the lower Lerma and upper Santiago rivers and their tributaries (Lyons et al. 1998). Since the late 1990s, *C. encaustus* has disappeared from the mainstem Santiago and Lerma rivers due to pollution and has become much less common in Lake Chapala owing to the invasion of the non-native *Poecilia sphenops* and *Gambusia yucatana* (Poeciliidae) (Becerra-Muñoz et al. 2003). *Chapalichthys encaustus* still persists in the lower portion of the Duero River drainage, a Lerma River tributary, including the La Luz and Orandino lakes, and also in Cajititlán and Los Negritos lakes, both near Lake Chapala. Beginning in 2005, small numbers of individuals have been collected from the La Vega Reservoir and its outlet in the upper Ameca River basin (Mar-Silva in press). These *C. encaustus* were probably introduced accidentally during a stocking of *Oreochromis aureus* (Cichlidae) from Lake Chapala or the Lerma basin.

*Chapalichthys pardalis*: Critically Endangered/Stable/2 ESUs (Figure 9) – This species is known from only two areas in the upper Balsas River basin, the San Juanico Lake and the Tocumbo Springs, located ca. 25 km downstream along the outlet of the lake (Miller et al. 2005). For many years the San Juanico population was considered a separate species, *C. peraticus* (e.g., Domínguez-Domínguez et al. 2005b), but recent genetic (Piller unpublished data) and morphological analyses (Miller et al. 2005) indicate that there are insufficient differences between the two populations to warrant separate species status, although they do qualify for separate ESU designation based on morphology. Chapa1, from Tocumbo, is critically endangered and possibly extinct in the wild. Formerly it was known only from a small spring system that had been heavily modified as a swimming area. However, none have been observed there since 2015. Chapa2, from San Juanico, is also critically endangered, having a small population occupying the nearshore areas of the lake.

***Characodon***: The distribution of this genus is separate from that of other Mexican goodeids, encompassing a portion of the Pacific slope of northwestern Mexico in the states of Durango and Coahuila far to the north of the other species (Figure 1). Two or possibly three species exist, one long extinct and the other(s) critically endangered and declining.

**Figure 11.**
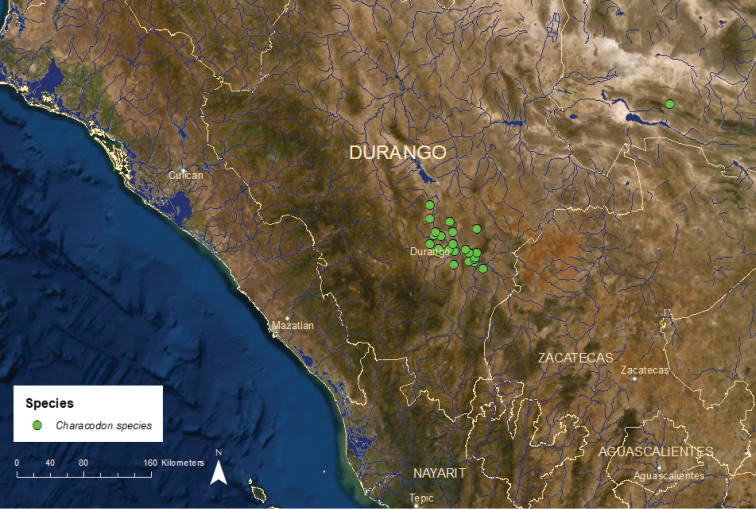
Distribution of *Characodon* species. The single record far to the east represents *C. garmani* and the remainder of points represent *C. audax* and *C. lateralis*.

*Characodon* species: Critically Endangered/Declining/9 ESUs (Figure 11) – The taxonomy and relationships of the *Characodon* populations occupying the upper Tunal and Durango river drainages in the upper Mezquital River basin in the state of Durango are currently unresolved. Originally, a single species, *C. lateralis*, was recognized, which occupied a series of semi-isolated spring systems near the city of Durango. However, the locality given for the original collection of the species, “Central America”, was clearly erroneous and the type material could not be attributed to a specific spring system (Miller et al. 2005). In the 1980’s, the population in the springs near the town of El Toboso was described as a separate species, *C. audax*, based on morphology, with the remaining populations considered *C. lateralis* (Smith and Miller 1986). However, more recent genetic analyses revealed little difference between the El Toboso population and other nearby *Characodon* populations (Doadrio and Domínguez-Domínguez 2004; Domínguez-Domínguez et al. 2006b). Instead, these analyses indicated that populations from spring systems located above the El Salto Waterfall on the Tunal River differed from those located below the falls, suggesting that perhaps all populations above the falls could be called *C. audax* and those below the falls *C. lateralis*. However, Artigas-Azas (2014) provided strong circumstantial evidence that the type of *C. lateralis* probably came from somewhere near the city of Durango above the falls, making it an inappropriate name for populations below the falls. Recent morphological analyses have indicated significant differences among ten populations, nine from above the falls and one from below, with the El Toboso population the most distinctive (Tobler and Bertrand 2014). Given uncertainly about which populations the name *C. lateralis* actually refers to and the discordance between the morphological and the genetic distinctiveness of the nominal *C. audax* from the El Toboso Springs, we have chosen to refer to all populations from the Tunal and Durango River drainages as “*Characodon* species”, pending a comprehensive revision of the genus. We have also identified nine ESUs, seven from above the falls and two from below, based on a combination of genetic, morphological, and zoogeographic information.

Regardless of what their taxonomic affinities are, all of the *Characodon* species ESUs are in serious trouble. Three ESUs have gone extinct in the last 20 years and the remaining six have all suffered steep drops in abundance (Artigas-Azas 2002, 2014). Declines have been caused by the drying of springs and streams owing to groundwater pumping and water diversions and by the introductions of non-native fish species. Chrsp1, the nominal *C. audax* from the El Toboso Springs, is critically endangered. Chrsp2, from the Cerro Gordo and El Carmen Springs and from the San Rafael and Las Moras streams, is also critically endangered and persists in small numbers only in the El Carmen Springs and in the Las Moras Stream in the town of San Rafael. Chrsp3, from the Los Pinos Springs and outlet, is extinct in the wild, with the last specimens collected in the late 1990’s. There are a few captive populations in Mexico, the United States, and Europe. Chrsp4, from the Guadalupe Aguilera, Laguna Seca, and Aguada de las Mujeres Springs and the Peñon del Aquila Reservoir, is critically endangered and current exists only in the Guadalupe Aguilera Springs. Chrsp5, from the San Vicente de los Chupaderos Springs and the Sauceda River, is extinct with no captive populations. The last collections date from the early 1990’s. Chrsp6, from the Abraham Gonzáles, Ojo Garabato, and 27 de Noviembre springs, is critically endangered but is still found in small numbers in all three springs. Chrsp7, from the Puente Pino Suárez Stream, is also critically endangered. Chrsp8, known from the Ojo de Aqua de San Juan, Los Berros, Ojo Nombre de Dios, and La Constancia springs, all located below the El Salto waterfall, is critically endangered and has disappeared from the Ojo Nombre de Dios Springs. Chrsp9, from the Amado Nervo Stream, also located below the El Salto Waterfall is probably extinct in the wild, with the last specimens observed in 2005. A small number of captive populations exists in Mexico, the United States, and Europe.

*Characodon garmani*: Extinct/No records since 1890’s/1 ESU (Chrga1) (Figure 3, 11) – This species is known from only a single female individual thought to have been collected from the endorheic Valley of Parras in Coahuila prior to 1895 (Fitzsimons 1972; Smith and Miller 1986; Miller et al. 2005), although there is circumstantial evidence that it may have come instead from a spring near Durango, making it a member of *Characodon* species rather than a separate taxon (Artigas-Azas, unpublished data). If it did indeed come from the Valley of Parras, it is no longer found there now and must be considered extinct with no captive populations (Miller et al. 1989). The habitat in Parras has been heavily modified during the last 150 years, and no specimens have been encountered during the many fish surveys conducted there from the 1940’s to the present.

***Girardinichthys***: This genus occurs in central Mexico in several different basins. Historically, *Girardinichthys* was believed to have included only two species, *G. multiradiatus* and *G. viviparus*. However, Radda and Meyer (2003) described a new species and combined the genus *Hubbsina* with *Girardinichthys* and relegated *Hubbsina* to a subgenus, which expanded *Girardinichthys* to four species. This change has not been accepted by some ichthyologists (e.g., Domínguez-Domínguez et al. 2005b; Miller et al. 2005).

**Figure 12.**
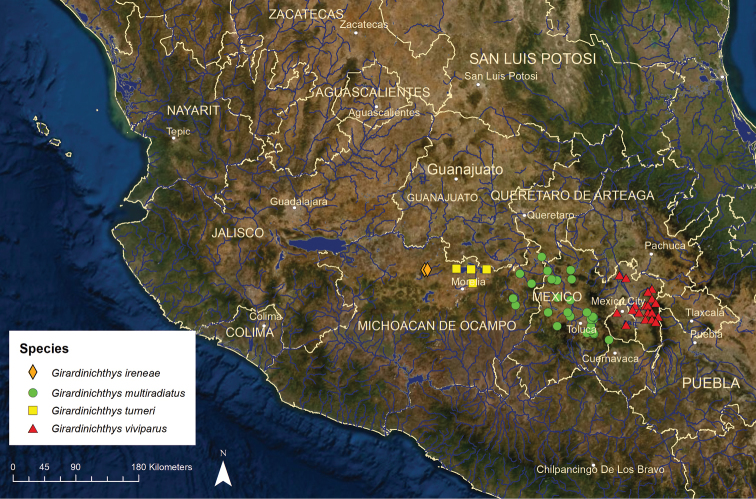
Distribution of the four species *Girardinichthys*.

*Girardinichthys ireneae*: Critically Endangered/Declining/1 ESU (Figure 12) – Until recently, this species was considered to be part of *Hubbsina turneri* (Domínguez-Domínguez et al. 2005; Miller et al. 2005). When Radda and Meyer (2003) subsumed *Hubbsina* within *Girardinichthys*, they split the former *H. turneri* into two species, *G. ireneae* and *G. turneri*. *Girardinichthys ireneae*, as currently defined, is known only from the upper portion of the Angulo River drainage of the Lerma River basin, primarily in Lake Zacapu and a few smaller spring-fed lakes nearby. It appears to have disappeared from the smaller lakes since 2000 and persists only in spring-fed areas of Lake Zacapu.

*Girardinichthys multiradiatus*: Endangered/Declining/2 ESUs (Figure 12) – This species was known historically from approximately 16 locations located just northwest, west, and south of greater Mexico City, including 13 streams and wetlands in the upper Lerma River basin and single sites in the headwaters of the Balsas River basin on the Pacific slope, the endorheic Lake Zempoala system, and the Taxingu Reservoir in the upper Pánuco River basin on the Atlantic slope (Domínguez-Domínguez et al. 2005b). Distribution and abundance of *G. multiradiatus* declined substantially in the Lerma basin during the 20^th^ Century, and the seven remaining populations there are now low in numbers and isolated, with some in decline and approaching extirpation. The Balsas, Zempoala, and Taxingu populations still persist but are small. Little clear genetic divergence is evident among populations from the Lerma, Balsas, and Pánuco basins (Macias-García et al. 2012). We used zoogeography to identify two ESUs. Girmu1 is limited to Lake Zempoala, which has been long isolated from the upper Lerma and Balsas basins and which experiences colder conditions than other goodeid habitats in Mexico. This ESU is endangered, with a moderate population in the small lake, which is fortunately protected as a National Park. Girmu2 encompasses all other populations and is also endangered.

Girardinichthys (Hubbsina) turneri: Extinct/No records since 1980s/1 ESU (Girtu1) (Figure 12) – As defined by Radda and Meyer (2003), this species was limited to Yuriria Lake in the Lerma River basin and the nearby endorheic Lake Cuitzeo/Grande de Morelia River basin. These two areas have been heavily polluted and modified, and no *G. turneri* have been observed in either area since the late 1980’s despite repeated and intensive targeted sampling (Soto-Galera et al. 1999; Domínguez-Domínguez et al. 2005b). Unfortunately, no captive populations exist, so this species appears to be extinct.

*Girardinichthys viviparus*: Endangered/Stable/1 ESU (Girvi1) (Figure 12) – Historically, this species was endemic to and abundant in the many lakes and wetlands of the endorheic Valley of Mexico, where Mexico City is located (Miller et al. 2005). Construction of a canal in the late 1800’s to drain the valley into the upper Tula River allowed the species to colonize Lake Zumpango and Lake Tecocomulco in the headwaters of the Pánuco River basin on the Atlantic slope. Lake Zumpango has poor water quality and the species may no longer exist there. Water quality in Lake Tecocomulco is good and *G. viviparus* persists there in moderate numbers. The drainage of the Valley of Mexico coupled with the rapid expansion of Mexico City led to a drastic decline in the distribution and abundance of *G. viviparus*. During the mid-20^th^ century, the species was eliminated from Lake Texcoco and Lake Chalco and became rare in Lake Xochimilco, all of which have become greatly reduced in size, highly polluted, and dominated by non-native fish species (Domínguez-Domínguez et al. 2005b). Despite poor environmental conditions, the Xochimilco population, which is very small, has managed to survive up to the present. Perhaps the largest remaining population is in the artificial Lake Lago Mayor in Chapultepec Park in downtown Mexico City. This population persists in moderate numbers and seems stable despite very poor water quality, but it is highly vulnerable to drainage of the lake for maintenance.

***Goodea***: This genus is now considered to have a single species (Miller et al. 2005; Domínguez-Domínguez et al. 2010) although in the past two or three species were recognized (e.g., Doadrio and Domínguez-Domínguez 2004). This genus is the most widespread of the Goodeids and occupies the broadest range of habitats, with a native distribution that encompasses most of the highlands of central Mexico including streams, rivers, wetlands, springs, lakes, and reservoirs (Miller et al 2005). This species is also, along with *Alloophorus robustus*, the largest of the goodeids and has been regularly used by humans for food.

*Goodea atripinnis*: Least Concern/Declining/2 ESUs (Figure 10) – This species has the largest distribution of any goodeid species. Its native range includes the Lerma, upper Santiago (including Lake Chapala), upper Ameca, upper Armería, and upper Balsas river basins on the Pacific slope, the endorheic Lake Zirahuén, Lake Pátzcuaro, and Lake Cuitzeo/Grande de Morelia River basins in central Mexico, and the upper Pánuco River basin on the Atlantic slope. Many years ago, *Goodea* was introduced and became established in the Valley of Mexico. Also, an introduced population was recently discovered in the upper Mezquital River basin within the range of *Characodon* near Durango (Michael Tobler, Kansas State University, Manhattan, Kansas, USA, unpublished data). Some early authors (e.g., Meek 1904; Mendoza 1962) considered the Lake Pátzcuaro population to be a different species, *G. luitpoldi*, but recent genetic and morphological analyses indicate that this population is not distinct from *G. atripinnis* (Doadrio and Domínguez-Domínguez 2004; Webb et al. 2004; Domínguez-Domínguez et al. 2010). Other authors have considered the Pánuco River basin population a distinct species, *G. gracilis* (e.g., Doadrio and Domínguez-Domínguez 2004; Domínguez-Domínguez et al. 2005b). Although there are minor genetic and morphological differences between Pánuco River basin populations and other *Goodea* populations, the Pánuco population is more appropriately considered as a separate ESU rather than a separate species (Domínguez-Domínguez et al. 2010).

*Goodea atripinnis* remains common in many areas and is probably still the most abundant goodeid species overall, but the distribution and abundance of both ESUs have steadily declined during the last 25 years (Lyons et al. 1998; Soto-Galera et al. 1999; Domínguez-Domínguez et al. 2005b; Mercado-Silva et al. 2006). Despite decreases in distribution and abundance, Gooat1 still qualifies as least concern and remains common in many areas. It appears to be relatively tolerant of poor water quality compared to other goodeids (Rueda-Jasso et al. 2017). Nonetheless, the trends for this ESU are not encouraging. Historically, this ESU supported commercial fisheries in the larger lakes where it occurred, but in recent years it has been eliminated from Lake Zirahuén, reduced to a small remnant population in Lake Pátzcuaro, and greatly decreased in number in Lake Chapala and Lake Cuitzeo, largely owing to predation by and competition with non-native fish species. It is still harvested and eaten in Lake Pátzcuaro and Lake Zacapu, Michoacán. Pollution and habitat modifications have devastated populations in many areas of the Lerma and upper Santiago basins. Gooat2 is endangered, and only four or five small populations persist in the upper Pánuco River basin. Decreases there have been caused primarily by water diversions and groundwater pumping, which have eliminated habitat.

***Ilyodon***: This genus is native to rocky, fast-flowing streams in the upper Ameca, Armería, Marabasco, Coahuayana, and Balsas basins in the mountains of west-central Mexico. It is most closely related to *Allodontichthys* and *Xenotaenia* (Doadrio and Domínguez-Domínguez 2004; Webb et al. 2004). Substantial morphological and genetic variation exists within and among populations of *Ilyodon* (Kingston 1979), and the taxonomy of the genus has long been confused. Based on recent genetic analyses (Beltrán-López et al. 2017), we recognize only two species, but some ichthyologists and aquarists have recognized as many as five or six.

**Figure 13.**
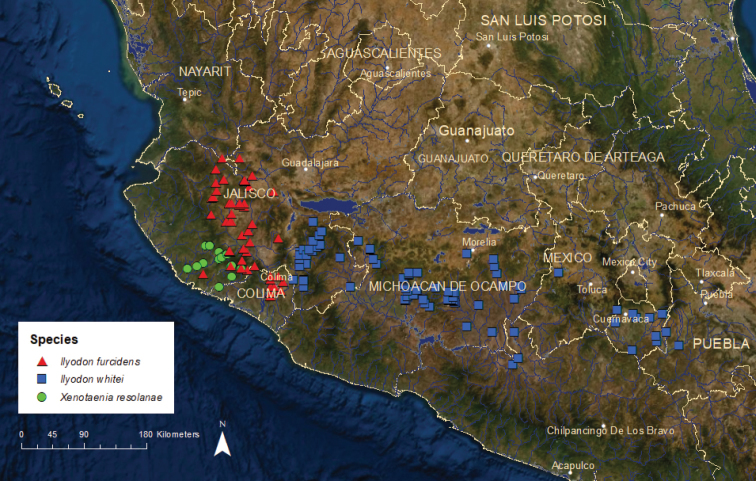
Distribution of *Ilyodon furcidens*, *I. whitei*, and *Xenotaenia resolanae*.

*Ilyodon furcidens*: Least Concern/Declining/1 ESU (Ilyfu1) (Figure 13) – As we define it, this species is widely distributed and common in the Armería basin and uncommon in the Marabasco and upper Ameca River basins. Historically, populations in the Coahuayana River basin were also assigned to this species (Miller et al. 2005), but recent genetic analyses indicate that those populations are distinct from Ameca, Marabasco, and Armería basin *I. furcidens* and better assigned to *I. whitei* (Beltrán-López et al. 2017). Populations in the upper Coahuayana basin have *I. whitei* morphology and appearance, whereas populations in the lower Coahuayana basin are more similar to *I. furcidens* in appearance and morphology, but both sets of populations are clearly distinct from *I. furcidens* (as we define it) genetically. Two morphotypes of *I. furcidens* are present in many areas of the Armería and Marabasco river basins (Lyons and Navarro-Pérez 1990), and these were long thought to be two different species, the narrow-mouthed form, *I. furcidens* and the wide-mouthed form, *I. xantusi*. However, work by Turner et al. (1983, 1985) and Grudzien and Turner (1984) demonstrated that narrow-mouthed females could produce both narrow-mouthed and wide-mouthed offspring, as could wide-mouthed females, proving that the two morphotypes were part of the same species. *Ilyodon furcidens* was the older of the two names and thus had priority, so the name *I. xantusi* is no longer considered valid. Some ichthyologists and aquarists consider populations from the upper Ameca River basin to be a separate species, *I. “amecae*” (Doadrio and Domínguez-Domínguez 2004). However, genetic and morphological differences between Ameca and Armería populations are small and more recent analyses do not consider the Ameca populations worthy of even separate ESU status (Beltrán-López et al. 2017). An introduced population of what appears to be *I. furcidens* was discovered in 2019 in the Citala Reservoir in the Lake Sayula basin (Köck, unpublished data).

*Ilyodon furcidens* is often the most common species at the localities where it occurs. It can reach a relatively large size (150 mm) and is sometimes consumed as food. However, numbers appear to be decreasing since 2000. Populations have declined or disappeared from several streams in the Ameca River basin because of shrinking water levels and invasions of non-native species. Within the Armería River basin, the expansion of the non-native *M. salmoides*, a top predator, and *Poeciliopsis gracilis* (Poeciliidae), a likely competitor and fry predator, has apparently resulted in the near elimination of *I. furcidens* from long stretches of the Ayuquila River, Jalisco (Lyons, unpublished data).

*Ilyodon whitei*: Vulnerable/Declining/5 ESUs (Figure 13) – As we define it, this species is found in the Coahuayana and Balsas river basins, where it occurs in ca. 60 sites over a wide range. Specimens from the Chacambero River, a tributary to the Balsas River near the town of Ciudad Altamirano in the state of Guerrero, were described as a separate species, *I. lennoni* (Meyer and Förster 1983), but based on limited and inconsistent morphological and genetic differences, we and most other ichthyologists (e.g., Doadrio and Domínguez-Domínguez 2004; Domínguez-Domínguez et al. 2005b; Miller et al. 2005; Beltrán-López et al. 2017) do not consider this species to be valid. Similarly, populations in Tacámbaro River drainage in the upper Balsas River basin were described as a separate species, *I. cortesae* (Paulo-Maya and Trujillo-Jiménez 2000), but again morphological and genetic differences between this and other populations are small and inconsistent, and we also do not consider this species to be valid.

Many populations of *I. whitei* have declined or disappeared during the last 25 years, largely because of predation by or competition from non-native fish species (e.g., Contreras-MacBeath et al. 1998) and water pollution. Non-native species now dominate many areas of the Balsas River basin. Of the five ESUs we recognize, Ilywh1, found in the upper Coahuayana River basin in Jalisco is vulnerable. It has declined from many areas but remains abundant where it occurs. Similarly, Ilywh2, distinguished from Ilywh1 based on morphology, from the lower Coahuayana basin in Jalisco, is also vulnerable. It too has declined but is still numerous in several areas. Ilywh3, in the central and western portion of the Balsas River basin and including the nominal *I. cortesae* and *I. lennoni*, is the most common and widespread ESU, occurring at ca. 30 sites, but still qualifies as vulnerable. Ilywh4 is classified as vulnerable and is found at ca. 12 sites in the Amacuzac River drainage in the eastern Balsas River basin in the state of Morelos. Ilywh5 is classified as vulnerable and has been reported from approximately ten sites in the Atoyac River drainage of the far eastern Balsas River basin in the state of Puebla.

***Neoophorus***: This genus currently consists of one species, *N. regalis*, which some ichthyologists place in the genus *Allotoca* (Webb et al. 2004; Miller et al. 2005). We concur with Doadrio and Domínguez-Domínguez (2004) that genetic information supports recognition of *Neoophorus* as a distinct genus, but additional genetic and morphological studies to confirm this view are warranted.

*Neoophorus regalis*: Critically Endangered/Declining/1 ESU (Neore1) (Figure 8) – At present, this species survives in only one small unnamed stream near the town of Los Reyes, Michoacán, in the upper Balsas River basin. Historically, the species was widespread and moderately common in streams and wetlands in the Valley of Los Reyes (Miller et al. 2005), but distribution and abundance have declined steadily during the last 25 years as wetlands have been drained and streams have been channelized and diverted for agriculture. Introductions of non-native *Xiphophorus helleri* (Poeciliidae) and *Oreochromis aureus* (Cichlidae) may also have contributed to losses. As of 2000, *N. regalis* was known from four locations (Domínguez-Domínguez et al. 2005b), but 2008 and 2011 surveys found the species at only one of these locations, where it was uncommon.

***Neotoca***: This genus consists of one species, *N. bilineata*, which many ichthyologists place in the genus *Skiffia* (Webb et al. 2004; Domínguez-Domínguez et al. 2005b; Miller et al. 2005). We concur with Doadrio and Domínguez-Domínguez (2004) that genetic information supports recognition of *Neotoca* as a distinct genus, but as in the case for *Neoophorus*, additional genetic and morphological studies to confirm this view are warranted.

**Figure 14.**
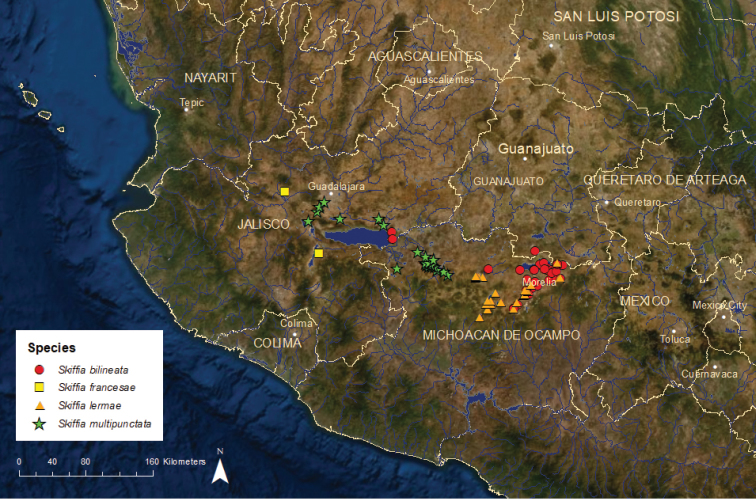
Distribution of *Neotoca bilineata* and the three species of *Skiffia*.

*Neotoca bilineata*: Endangered/Declining/2 ESUs (Figure 14) – Historically, this species was reported from three distinct areas: Lake Chapala and adjacent portions of its outlet, the upper Santiago River; the Lerma River and its tributaries the Laja River, Turbio River, and Lake Yuriria in the middle Lerma River basin; and the endorheic Lake Cuitzeo/Grande de Morelia River basin near the city of Morelia (Miller et al. 2005), but it has declined dramatically throughout its range. We recognize two ESUs based on genetic analyses (Ornelas-García et al. 2012). Neobi1, from Lake Chapala and vicinity, appears to be extinct with no captive populations. Most records are from the early 1900s, and no individuals have been collected there for at least 70 years (Lyons et al. 1998). Neobi2 is critically endangered and occupies the rest of the species’ range. Only a remnant population remains in the middle Lerma River near the city of Salamanca and in two small springs tributary to the Turbio River near Penjamo, and the species has been lost from the Laja River drainage and Lake Yuriria (Soto-Galera et al. 1998; Mercado-Silva et al. 2006; Mercado and Piller unpublished data). Populations persist in the Lake Cuitzeo/Grande de Morelia River basin in Lake Cuitzeo, Cointzio Reservoir, Querendaro River, and Borbollon Springs, but none of these are particularly large and numbers fluctuate greatly within and among years. Pollution and habitat modifications had eliminated the species from nearly all of Lake Cuitzeo proper and from most of the Grande de Morelia River drainage before 2000 (Soto-Galera et al. 1999). Population declines have continued since then, and the long-term survival of this species in the wild is uncertain (De la Vega-Salazar et al. 2003a; Domínguez-Domínguez et al. 2005b).

***Skiffia***: As we define it, this genus has three species, all limited to central and west-central Mexico where they are found mainly in springs and spring-fed lakes and streams (Miller et al. 2005).

*Skiffia francesae*: Extinct in the Wild/No records since 2008/2 ESUs (Figure 14) – This species had long been thought to be endemic to the Teuchitlán Springs in the upper Ameca River basin and was already believed extinct in the wild when it was first formally described as a species in 1978 (Kingston 1978; Miller et al. 1989). Fortunately, captive populations exist in North America and Europe, although many of these are inbred (Domínguez-Domínguez et al. 2005b). In 2007, Omar Domínguez-Domínguez discovered a new population of *Skiffia* that suggested that *S. francesae* might still persist in wild. These fish were found at the El Molino spring pond near the town of Cuyucapán in the endorheic Lake Sayula basin, which is located ca. 80 km south of the Teuchitlán Springs. Some individuals from El Molino appeared identical to *S. francesae*, whereas others had pigmentation that was more reminiscent of *S. multipunctata*. Genetically, the El Molino fish were more similar to *S. francesae* than to *S. multipunctata*. Consequently, we have chosen to treat the two populations as separate ESUs of *S. francesae*, Skifr1 for Teuchitlán and Skifr2 for El Molino. Unfortunately, the El Molino population was eliminated when the pond dried completed during a drought in 2010. Thus, both ESUs are extinct in the wild. Moderate numbers of captive populations of Skifr1 exist in Mexico, the United States, and Europe, but captive populations of Skifr2 are scarce.

*Skiffia lermae*: Endangered/Declining/4 ESUs (Figure 14) – The historical range of this species encompassed many sites in central Mexico including Lake Zacapu, Lake Yuriria, and the Laja River in the middle Lerma River basin, and the endorheic Lake Pátzcuaro, Lake Zirahuén, and Lake Cuitzeo/Grande de Morelia River basins. Distribution and abundance of *S. lermae* have declined steadily during the last 50 years, due to water pollution, habitat degradation, and non-native species, with continued losses through the 2000s. The species has disappeared from nearly all of the Laja River drainage, Lake Yuriria, Lake Cuitzeo, and the entire Lake Zirahuén basin, and has become uncommon and limited to Lake Zacapu and a few small springs in the Lake Pátzcuaro and Grande de Morelia River basins (Lyons et al. 1998; Soto-Galera et al. 1998, 1999; De la Vega-Salazar 2003a; Domínguez-Domínguez et al. 2005b, 2008a; Mercado-Silva et al. 2006). Four ESUs are recognized, all in trouble. Skile1 occupies Lake Zacapu in the middle Lerma River basin where it is endangered. Skile2 has been reported from Lake Yuriria and the endorheic Lake Cuitzeo/Grande de Morelia River basin and is endangered. Populations from Lake Yuriria and Lake Cuitzeo are gone, but this ESU persists in the La Mintzita Springs, tributary to the Grande de Morelia River. Skile3 is known from the endorheic Lake Zirahuén and Lake Pátzcuaro basins and is also endangered. This ESU has been eliminated from Lake Zirahuén and Lake Pátzcuaro but persists in the Molino de Chapultepec Springs in the Lake Pátzcuaro basin. Skile4 is known from the Laja River drainage and is critically endangered. Historically this ESU was common throughout the drainage but now it is restricted to the Charco del Ingenio Reserve on the De Las Colonias Reservoir in the city of San Miquel de Allende, Guanajuato.

*Skiffia multipunctata*: Endangered/Declining/1 ESU (Figure 14) – This species was found historically in Lake Chapala, the upper part of the Santiago River basin near the city of Guadalajara, including Lake Cajititlán, and the lower Lerma River basin, particularly the Duero River drainage (Domínguez-Domínguez et al. 2005b). Pollution, habitat modifications, and introductions of non-native species have eliminated *S. multipunctata* from Lake Chapala, the Santiago River basin, and parts of the lower Lerma River basin (Lyons et al. 1998; Soto-Galera et al. 1998). The only area where the species remains is the Duero River drainage, but the species has disappeared from the lower portion of the drainage because of stream channelization and water diversions for agriculture. Only six or seven populations remain, with the largest found in the spring-fed La Luz and Orandino lakes. Populations in both lakes are threatened by habitat modifications for recreation and introductions of non-native fishes. Information on the larval ecology of *S. multipunctata* in captivity is provided by Escalera-Vázquez et al. (2004) and Kelley et al. (2005).

***Xenoophorus***: As presently defined, this genus has only one species, *X. captivus*, which is known from three hydrologically and geographically isolated areas on the Atlantic slope in the state of San Luis Potosí that are located to the northeast of the main body of the overall goodeid range (Miller et al. 2005). Populations from each area are somewhat distinctive morphologically and genetically, and they were formerly considered three different species until synonymized as one by Fitzsimons (1979). We consider them three separate ESUs based on their genetic and morphological characteristics.

*Xenoophorus captivus*: Endangered/Declining/3 ESUs (Figure 10) – The distribution and abundance of this species has shrunk considerably since the 1970’s and 1980’s owing to groundwater pumping and spring diversions that have lowered water levels and degraded water quality. Of the three ESUs, Xenca1 is critically endangered. Two or three small and somewhat interconnected populations are known from the upper portion of the Santa María del Río drainage in the upper portion of the Pánuco River basin in southern portion of the state of San Luis Potosí. Xenca2 is extinct in the wild. Historically it was found in the endorheic Illescas spring system near the border of the states of Zacatecas and San Luis Potosí, with the last confirmed collection from 1994 (Artigas-Azas 1995). A few captive populations exist in Mexico, the United States, and Europe. Xenca3 is endangered. Populations were known from three small springs, Venados, Moctezuma, and Agua de Enmedio, located in a small endorheic basin in the northern part of San Luis Potosí. These three populations still exist but are small.

***Xenotaenia***: This genus has a single species, *X. resolanae*, limited to streams and small rivers in the Marabasco and Purificación river basins on the Pacific slope of west-central Mexico. The Marabasco and Purificación populations differ morphologically (Lyons 1996), and we consider them separate ESUs. The species is most closely related to *Allodontichthys* and *Ilyodon* (Doadrio and Domínguez-Domínguez 2004; Webb et al. 2004; Miller et al. 2005).

*Xenotaenia resolanae*: Vulnerable/Stable/2 ESUs (Figure 13) – This species is known historically from a total of ca. 20 locations. Xenre1, which occupies the upper Marabasco River basin, is vulnerable, and occurs at approximately ten locations. The populations, while small, seem to be stable (Lyons and Navarro-Pérez 1990; Lyons 1996; Domínguez-Domínguez et al. 2005b; Lyons unpublished data). Xenre2, which occupies ca. ten locations in the upper Purificación River basin, is also vulnerable. Water pollution from sugar mill discharges, human sewage, and animal wastes had eliminated or reduced several populations by the 1980s, but since then the remaining populations seem to have stabilized.

***Xenotoca***: Based on the morphological analyses of Fitzsimons (1972) and the genetic and morphological analyses of Domínguez-Domínguez et al. (2016), this genus is currently considered to have five species, the long-established *X. eiseni*, *X. melanosoma*, and *X. variata*, and the recently described *X. doadrioi* and *X. lyonsi* (Domínguez-Domínguez et al. 2016). However, Webb (1998), in his Ph.D. dissertation, provided genetic and morphological evidence that *X. eiseni* and *X. melanosoma* are not the closest relatives of *X. variata*. This conclusion was supported by further genetic analyses by Doadrio and Domínguez-Domínguez (2004), Webb et al. (2004), and Domínguez-Domínguez et al. (2016). Webb (1998) proposed that *X. variata* remain in *Xenotoca*, and the remaining species be placed in a new and separate genus “*Xenotichthys*”. Consequently, in some publications, the two species were referred to as “*Xenotoca*” *eiseni* and “*Xenotoca*” *melanosoma* to indicate the likelihood that their genus would eventually change (e.g., Miller et al. 2005). To date, the proposal by Webb (1998) to apply “*Xenotichthys*” as the genus for *X. eiseni* and *X. melanosoma* has not yet been formally published.

**Figure 15.**
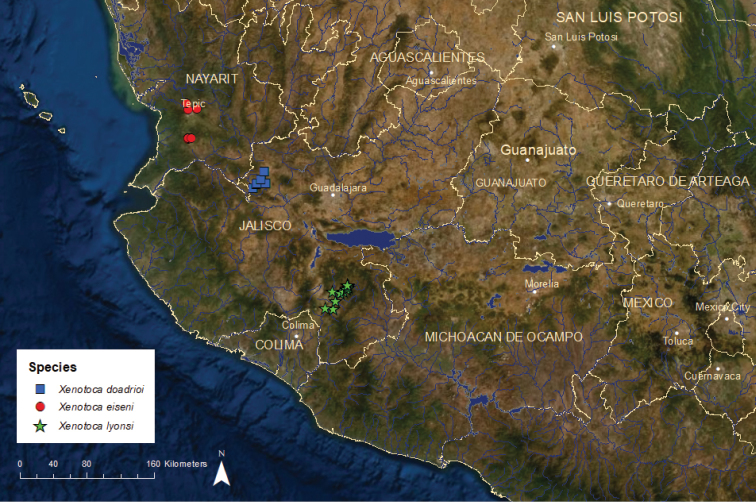
Distribution of *Xenotoca doadrioi*, *X. eiseni*, and *X. lyonsi*.

*Xenotoca doadrioi*: Endangered/Declining/1 ESU (Xendo1) (Figure 15) – This species was shown to be genetically distinctive by Piller et al. (2015) and was recently separated from *X. eiseni* (Domínguez-Domínguez et al. 2016). It is found in the San Marcos drainage of the upper Ameca River Basin and the adjacent endorheic Lake Magdalena basin. Since 2000, it has disappeared from many locations due to water diversions and groundwater pumping that have eliminated many springs and small streams, and probably also from competition with or predation by the non-native *Pseudoxiphophorus bimaculatus* (Poeciliidae), which appears to have displaced *X. doadrioi* in some places. Only a handful of populations of *X. doadrioi* persist.

*Xenotoca eiseni*: Endangered/Declining/2 ESUs (Figure 15) – This species was recently split into three: *Xenotoca doadrioi*, *X. eiseni*, and *X. lyonsi* (Domínguez-Domínguez et al. 2016). As currently defined, *X. eiseni* is known from the upper Santiago River basin near the city of Tepic, and the upper portions of direct Pacific Ocean drainages near the city of Compostela, all in the state of Nayarit. We recognize two ESUs based on genetic differences. Xenei1 is endangered and is found in the Santiago River basin near the city of Tepic. Many populations have disappeared owing to lack of water, habitat destruction, and introductions of non-native species, and only a handful of small populations remain. Xenei2 is also endangered and known from the direct drainages to the Pacific Ocean near the city of Compostela. It too has declined dramatically for the same reasons as Xenei1 and survives in small numbers in just a few springs and streams.

*Xenotoca lyonsi*: Endangered/Declining/1 ESU (Xenly1) (Figure 15) – This species was recently separated from *X. eiseni* (Domínguez-Domínguez et al. 2016). It is known from the upper Coahuayana River basin and is endangered. Historically it was widespread, but lack of water and water pollution have eliminated most populations.

**Figure 16.**
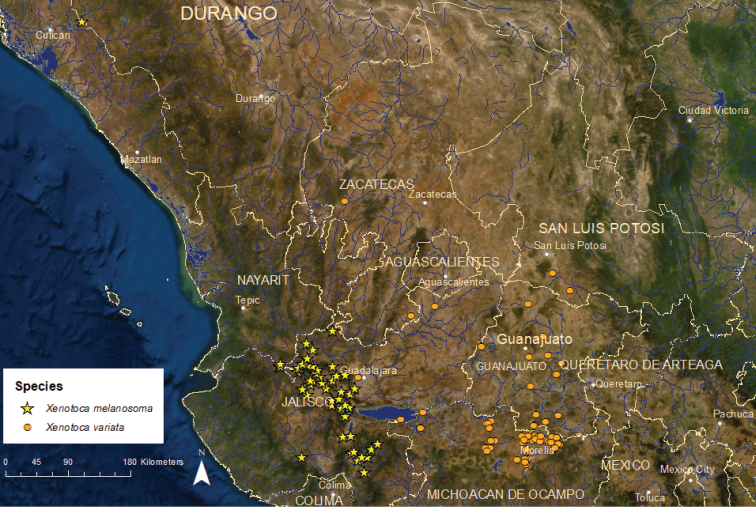
Distribution of *Xenotoca melanosoma* and *X. variata*.

*Xenotoca melanosoma*: Vulnerable/Declining/4 ESUs (Figure 16) – This species is found in the Santiago, Ameca, Armería, and Coahuayana river basins and the endorheic Magdalena, Atotonilco, San Marcos, Zacoalco, Sayula, and Zapotlán lake basins in Jalisco (Miller et al. 2005). We recognize four ESUs based on genetics and zoogeography (Domínguez-Domínguez et al. 2010; Mar-Silva 2018). Xenme1 is by far the most numerous and widely distributed and is classified as vulnerable. It is found in the Ameca, Magdalena, Atotonilco, San Marcos, Zacoalco, and Sayula basins at a total of ca. 15 locations. It has declined since 2000 owing to water pollution, habitat degradation, and non-native species. It has disappeared from the San Marcos and Zacoalco basin and persists at only 1–3 locations in the Magdalena, Atotonilco, and Sayula basins. The best remaining populations are in the Ameca basin. Xenme2 is critically endangered and is known from Lake Zapotlán where it is rare and in decline from habitat modifications and non-native species. Xenme3 is endangered and currently known from two locations on the upper Tamazula River where it is threatened by water diversions and introduced species. Xenme4 is also endangered and is limited to a short reach of the Ayuquila River downstream of the city of El Grullo in the Armería River basin, where chronic water quality degradation limits the population.

*Xenotoca variata*: Least Concern/Declining/5 ESUs (Figure 16) – This species is broadly distributed in central Mexico throughout the Lerma and upper Santiago river basins on the Pacific slope, the endorheic Lake Cuitzeo/Grande de Morelia River basin in central Mexico, and a small area of the upper Pánuco River basin on the Atlantic slope (Miller et al. 2005). It reaches a relatively large total length (~100 mm) and is caught and eaten in Zacapu Lake, Michoacán. It is highly tolerant of pollution and habitat modifications and, along with *Goodea atripinnis*, still persists in areas where other goodeid species have been eliminated. *Xenotoca variata* is currently found at many locations throughout its historic range. Nonetheless, the species has declined in recent years, disappearing from heavily polluted areas of the Santiago and Lerma basins and from reservoir and lake habitats where the non-native *Micropterus salmoides* (Centrarchidae) has become established (Lyons et al. 1998; Soto-Galera et al. 1998, 1999; Domínguez-Domínguez et al. 2005b; Mercado-Silva et al. 2006). We recognize five ESUs based on genetic analyses (Domínguez-Domínguez et al. 2010). Xenva1 is classified as least concern and broadly distributed in the Santiago, Lerma, and Pánuco basins. Many populations are still present, but others have disappeared from the Lerma River and its major tributaries due to water pollution. Xenva2 is vulnerable and known from a single location, Lake Los Negritos (also known as La Alberca). The population there remains moderately large but appears to have declined because of non-native species. Xenva3 is vulnerable and found in the Angulo River drainage in the middle Lerma River basin. It has declined in abundance in the river but remains relatively common in the headwaters at Lake Zacapu. Xenva4 is vulnerable and known from the endorheic Aquanaval River basin. It has declined because of overuse of water and habitat loss. Xenva5 is least concern and found in Lake Cuitzeo and the Grande de Morelia River basin. It has declined in the lake proper owing to habitat loss and poor water quality and has been eliminated from the Grande de Morelia River near the city of Morelia, but still remains common at several locations. This last ESU is particularly distinctive genetically and may eventually be described as a separate species.

***Zoogoneticus***: This genus is found over a large portion of central and west-central Mexico. Until the late 1990’s, *Zoogoneticus* was thought to have only one species, the wide-ranging *Z. quitzeoensis*. Then in 1998, *Z. tequila* was described (Webb and Miller 1998) and in 2008, *Z. quitzeoensis* was split into two species, *Z. quitzeoensis* and *Z. purhepechus* (Domínguez-Domínguez et al. 2008b).

**Figure 17.**
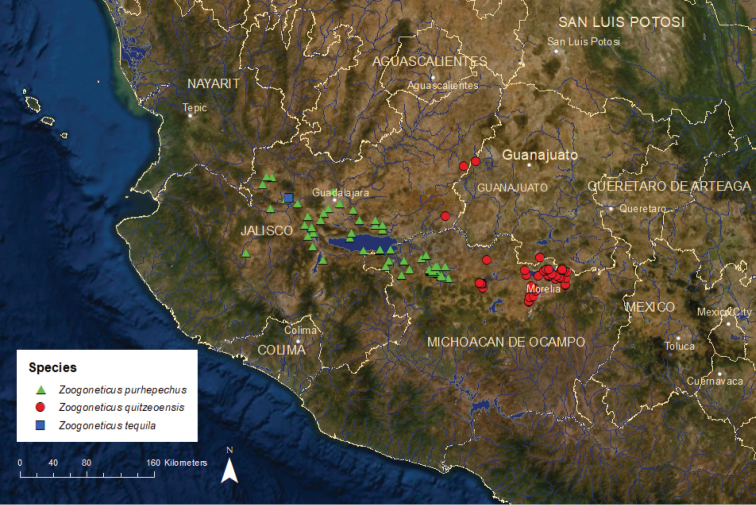
Distribution of the three species of *Zoogoneticus*.

*Zoogoneticus purhepechus*: Vulnerable/Declining/3 ESUs (Figure 17) – This species was recently separated from *Z. quitzeoensis* based on genetic and morphological differences (Domínguez-Domínguez et al. 2007, 2008b). The historical range of this species, as currently defined, encompassed the lower Lerma, upper Santiago (including Lake Chapala), upper Ameca, and upper Armería river basins on the Pacific slope, and the endorheic Lake Magdalena, Atotonilco, San Marcos, and Sayula basins in west-central Mexico (Miller et al. 2005; Domínguez-Domínguez et al. 2008b). Lake drying, water pollution, and introductions of non-native species have eliminated *Z. purhepechus* from many areas. We recognize three ESUs based on genetics and zoogeography. Zoopu1 is vulnerable and occurs in the upper Santiago and lower Lerma River basins including Lake Chapala. It has become scarce in Lake Chapala due to non-native species and has disappeared from much of the Santiago and Lerma basins owing to water pollution and habitat destruction. The best remaining populations occur in four springs that drain to the Duero River, a tributary of the Lerma River (Lyons et al. 1998; Soto-Galera et al. 1998; Moncayo-Estrada et al. 2015). Zoopu2 is endangered and is known from the Upper Ameca River basin and the endorheic Lago Magdalena basin. Once moderately common, it is now rare at one location in the Magdalena basin and occurs at only one or two locations in the Ameca basin, declining because of water pollution, habitat loss, and non-native species. Zoopu3 is critically endangered and known historically from the Armería River and Atotonilco and San Marcos lake basins. Habitat destruction, water diversions, water pollution, and non-native species have eliminated this ESU from the Armería and Atotonilco basins, and it persists in very small numbers at one site in the San Marcos basin (Lyons et al. 1998; Domínguez-Domínguez et al. 2008b; Domínguez unpublished data).

*Zoogoneticus quitzeoensis*: Endangered/Declining/2 ESUs (Figure 17) – As currently defined, this species was known historically from the Angulo, Turbio, and Laja river drainages and Lake Yuriria in the middle Lerma River basin, and from throughout the endorheic Lake Cuitzeo/Grande de Morelia basin in central Mexico (Domínguez-Domínguez et al. 2007, 2008b). Since 2000, *Z. quitzeoensis* has disappeared from many areas owing to a combination of water pollution, habitat loss from water diversions, and introduction of non-native species (Lyons et al. 1998; Soto-Galera et al. 1997, 1998; De la Vega-Salazar et al. 2003a, De la Vega-Salazar and Macías-Garcia 2005; Domínguez-Domínguez et al. 2005b, 2008a; Mercado-Silva et al. 2006). We recognize two ESUs based on genetic analyses (Domínguez-Domínguez et al. 2008b). Zooqu1 is critically endangered and was known historically from the Laja and Turbio river drainages. Populations in the Laja are now gone, and nearly eliminated from the Turbio. One or two populations may still persist in springs draining to the Turbio River. Zooqu2 is endangered and known historically from Lake Yuriria, the endorheic Lake Cuitzeo/Grande de Morelia basin, and the Angulo River drainage. The populations in Lake Yuriria, Lake Cuitzeo, and the Grande de Morelia River have been eliminated, and the species persists at only a few locations. The best remaining populations are in Lake Zacapu at the headwaters of the Angulo River drainage, and La Mintzita Springs, which drains to the Grande de Morelia River near Morelia.

*Zoogoneticus tequila*: Extinct in the Wild/Last Record 2008/1 ESU (Figure 17) – This species, endemic to the Teuchitlán Springs in the upper Ameca River basin in the state of Jalisco, was thought to be already extinct in the wild when it was formally described in 1998 (Webb and Miller 1998; Miller et al. 2005). However, in 2000, a tiny remnant wild population was discovered in a small and isolated area of the Teuchitlán Springs (De la Vega-Salazar 2003b; Domínguez-Domínguez et al. 2005b, 2008a). This population was so small (< 100 individuals) that it was already inbred (Bailey et al. 2007). The last collection from there was in 2008. A major drought in 2010 completely dried the habitat of *Z. tequila*, and when the drought ended and water levels increased, the area was invaded by the non-native *Pseudoxiphophorus bimaculatus* (Poeciliidae), a species that has been associated with the decline of several goodeid species. Many efforts to find *Z. tequila* were undertaken after 2010 without success and eventually the species was declared truly extinct in the wild. Fortunately, captive populations are relatively common in Mexico, the United States, and Europe. In 2014, faculty, students, and staff at the Universidad Michoacana de San Nícolas de Hidalgo, in Morelia, state of Michoacán, began a project to re-introduce captive stocks of *Z. tequila* into the Teuchitlán Springs. They exposed the captive fish to semi-wild conditions in outdoor ponds for several generations and then in 2016 added pond fish to a different semi-isolated area of the springs from which nearly all non-native species had been removed. Thus far the stocked fish are surviving and reproducing. However, continued monitoring and removal of non-native species will probably be required to ensure that *Z. tequila* persists in the Teuchitlán Springs.

## Discussion

The Mexican goodeids are at a crossroads. Once the most diverse, widespread, and numerous fishes in central Mexico (Miller et al. 2005), they now have been reduced to only a shadow of their former distribution and abundance. If they are to survive the 21^st^ century, or even the next few decades, major conservation initiatives must be undertaken. We believe three steps are essential. First, the best remaining habitats for each species and ESU should be protected. For those goodeids that are able to live in springs or the standing water of small spring-fed lakes, habitat protection may be a practical goal. Many of the springs and spring-fed lakes containing the most viable remaining goodeid populations are already formally or informally protected as municipal or even national parks or as sources of water for drinking and irrigation. These designations do not fully protect them from habitat modifications for recreation, agriculture, or water extraction, or reductions in the water table caused by regional groundwater pumping (e.g., Scott and Shah 2004) or climate-change-induced severe droughts (e.g., Mulholland et al. 1997). The local and national governmental entities charged with managing them sometimes work at cross purposes, but at least these springs and lakes have groups likely to advocate for their conservation. A more insidious problem is non-native species. In many springs and lakes of central Mexico, non-native fish species such as *Cyprinus carpio* (Cyprinidae), *Ictalurus punctatus* (Ictaluridae), *Oncorhynchus mykiss* (Salmonidae), *Micropterus salmoides* (Centrarchidae), or *Oreochromis aureus* (Cichlidae) have been stocked to provide a food source (Lyons et al. 1998; Moncayo-Estrada et al. 2012; Gesundheit and Macías-García 2018). Smaller-bodied non-native fish species such as poeciliids often arrive as contaminants from these stockings or as escapees or direct releases of aquarium fish (Contreras-MacBeath et al. 1998; Lyons et al. 1998; Moncayo-Estrada et al. 2012, 2015; Gesundheit and Macías-García 2018). The interactions of goodeids with non-native species are generally poorly documented or understood (Ramírez-Carrillo and Macías-García 2015; Ramírez-García et al. 2018), but in almost all cases the establishment of one or more non-natives is associated with declines in abundance of the goodeids (e.g., Soto-Galera et al. 1998, 1999; Gesundheit and Macías-García 2018). Introductions of non-native species have been and will likely continue, and once established, non-native species are difficult to eradicate, and long-term goodeid preservation may be challenging even in undegraded and well-protected springs.

For those goodeids requiring flowing waters (e.g., *Allodontichthys*, *Ilyodon*, and *Xenotaenia*) a much larger area of land may need to be managed to encompass the watershed of the stream or river occupied by the goodeids. Most parks or protected areas will be insufficient by themselves, but in concert with less restrictive land management schemes, such as biosphere reserves (Batisse 1997), it may be possible to provide at least partial protection from major water diversions, industrial or municipal pollution, and habitat destruction. For example, the Sierra de Manantlán Biosphere Reserve in the state of Jalisco helps implement land and water management practices that conserve populations of *Allodontichthys zonistius*, *Ilyodon furcidens*, and *Xenotaenia resolanae* (Lyons and Navarro-Pérez 1990). However, some stream and river-dwelling goodeid species and ESUs have essentially no protected or sustainably managed land in their watersheds, and their future survival is uncertain.

Second, where practical, degraded habitats should be rehabilitated. The key is choosing habitats where some recovery of goodeid populations is realistic through re-colonization or re-introduction once habitat quality is improved. Many habitats in central Mexico have been completely obliterated or are so modified that goodeid restoration is infeasible (Lyons et al. 1998; Soto-Galera et al. 1998, 1999; Mercado-Silva et al. 2006; Moncayo-Estrada et al. 2015). However, where only a single type of habitat or water quality/quantity degradation limits the goodeid population, recovery may be possible. For example, a 50-km segment of the Ayuquila River in the Upper Armería Basin, had been rendered fishless from severe water pollution from a sugar mill (Lyons et al. 1998). Fortunately, goodeids and other species persisted further downstream and in tributaries. Diversion of the sugar mill wastes into irrigation canals for several kilometers allowed for partial breakdown of the wastes, and water quality in the river improved. *Ilyodon furcidens*, a relatively tolerant goodeid, and two tolerant native poeciliids were able to re-colonize most of the 50-km segment, avoiding only the 10-km stretch immediately below the discharge of the sugar mill wastes into the river from the irrigation canals. As a second example, in the Teuchitlán Springs, habitat and water quality and quantity remained adequate for goodeid survival, but abundant non-native species had eliminated two species. Manual reduction of non-native fish populations to low levels allowed for the successful re-introduction of *Zoogoneticus tequila*.

Third, and finally, captive populations of rare goodeid species and ESUs should be established. Relying on only protecting and restoring wild populations is too risky. Many goodeid species and populations face such daunting environmental challenges that they will likely disappear soon from the wild even with the best-possible on-the-ground conservation efforts. Fortunately, most goodeids can be maintained and bred relatively easily in captivity. Currently, two Mexican universities, the Universidad Michoacana de San Nícolas de Hidalgo (UMSNH) in Morelia, Michoacán, and the Universidad Autónoma de Nuevo León (UANL) in Monterrey, Nuevo León, maintain goodeids. The UMSNH has nearly all of the extant goodeid species and the UANL has a selection of some of the rarest species. Several zoos and public aquariums in the United States and Europe also maintain one or more goodeid species. But collectively, the total holdings of all public institutions in Mexico, the United States, and Europe do not cover all of the goodeid ESUs, and many species are represented by a single population at a single institution, vulnerable to accidental or catastrophic loss. Funding and technical support for goodeid conservation at many institutions is also limited and uncertain for the future.

We believe that a key component of goodeid captive maintenance is participation by aquarium hobbyists (Maceda-Veiga et al. 2014). A relatively small but passionate group of hobbyists already maintains goodeids, and we are working to increase their numbers and holdings. We have developed a framework and network to facilitate goodeid conservation by aquarists through the Goodeid Working Group (GWG: http://www.goodeidworkinggroup.com/). This voluntary organization of scientists, conservationists, and hobbyists provides a mechanism for exchanges of information, promotion of good conservation principles in captive maintenance, and fund-raising to support goodeid conservation in Mexico. Communication is facilitated through a website, regular email and Facebook updates, and annual face-to-face meetings in Mexico, the United States, and Europe that allow hobbyists and goodeid scientists and conservationists to interact. Hobbyists often have important data to share on the husbandry and behavior of goodeid species. The captive populations that hobbyists maintain can also be a source for re-introductions in the wild. Indeed, the *Zoogoneticus tequila* that were recently re-introduced into the Teuchitlán Springs were ultimately derived from a captive population maintained by an English aquarist, the late Ivan Dibble. He kept the species for many years and returned live specimens to the UMSNH in the late 1990’s.
